# Supplementary material for: Implementation of the ERAS (Enhanced Recovery After Surgery) protocol for hysterectomy in the Piedmont Region with an audit&feedback approach: Study protocol for a stepped wedge cluster randomized controlled trial. A study of the EASY-NET project
Source: PLoS One. 2022 May 27;17(5):e0268655. doi: 10.1371/journal.pone.0268655 (PMC9140274; doi:10.1371/journal.pone.0268655)
Supplement: S1 File — (DOCX) [file pone.0268655.s001.docx]

**Supporting Information – Implementation of the ERAS (Enhanced Recovery After Surgery) protocol for hysterectomy in the Piedmont Region with an audit&feedback approach: study protocol for a stepped wedge cluster randomized controlled trial. A study of the EASY-NET project**

**Contents:**

**- S1 Table. ERAS protocol items for gynecologic surgery – pag 2**

**- Synoptical scheme of the protocol – pag 3**

**- Statistical plan – pag 7**

**- Data collection – pag 9**

**- SPIRIT check list – pag 10**

**- RECOvER checklist – pag 15**

**Table S1. ERAS protocol items for gynecologic surgery.**

| *Preoperative items* | - Preadmission counselling - No prolonged fasting - Carbohydrate loading (oral carbohydrate drinks up until 2 hours, before initiation of anaesthesia) - No bowel preparation - Thromboembolism prophylaxis - Antibiotic prophylaxis - No premedication |
| --- | --- |
| *Intraoperative items* | - Prevention of hypothermia (body warmer/warm intravenous fluids) - Restrictive intraoperative fluid management strategy - Adoption of minimally invasive surgical techniques when feasible - Surgical drainage only in selected cases |
| *Postoperative items* | - Multimodal analgesia (minimized opioid use) - Prevention of nausea and vomiting - No nasogastric tubes - Early removal of urinary catheter - Early removal of intravenous infusions - Early re-feeding - Early mobilization - Criteria for discharge - Feedback about compliance and outcomes |

**Synoptical scheme of the protocol**

| **Descriptive information** |  |
| --- | --- |
| **ClinicalTrials.gov Identifier** | NCT04063072 |
| **Brief Title** | Audit and feedback approach to implement the ERAS protocol for hysterectomy in the Piedmont Region |
| **Official Title** | Implementation of the ERAS (Enhanced Recovery After Surgery) protocol for hysterectomy in the Piedmont Region with an audit and feedback approach: study protocol for a stepped wedge cluster randomized controlled trial. A study of the EASY-NET project |
| **Brief Summary** | To promote the systematic adoption of the ERAS perioperative program for hysterectomy  in the entire regional hospital network in Piedmont (North-West of Italy), an Audit-and-Feedback approach (A&F) has been adopted within a cluster randomized controlled trial aiming to estimate the true impact of the protocol on a large, unselected population. Every hospital is a cluster entering the study treating patients according to its current clinical practice. On the basis of a randomized order, each hospital switches from current clinical practice to the adoption of the ERAS protocol. |
| **Detailed Description** | ERAS (Enhanced Recovery After Surgery) protocol is a multimodal perioperative care pathways designed to achieve early recovery after surgical procedures by maintaining preoperative organ function and reducing the stress response following surgery. Even if efficacy and safety of ERAS protocol in gynaecological surgery is well-established in the literature, its implementation is limited to few selected centres in Piemonte. The aim of the study is to extend the implementation of the ERAS protocol to whole regional network of hospitals. Specific objectives are to estimate its impact on different dimensions of quality of care, including length of stay, complications and patient satisfaction, and to identify possible barriers or facilitating factors. |
| **Study Type** | Interventional |
| **Study Design** | Allocation: Randomized Intervention Model: Crossover Assignment Intervention Model Description: A stepped-wedge cluster randomized clinical trial. Each center starts as control group (usual care) and switches to experimental group (ERAS protocol implementation), according to a randomized order.  Masking: None (Open Label) Primary Purpose: Health Services Research |
| **Intervention** | Procedure: ERAS protocol  In gynecological surgery, the ERAS protocol involves an accurate interview with the patient in the preoperative phase aimed at smoking and alcohol cessation, the reduction of preoperative fasting, the omission of intestinal preparation, the prophylaxis of thromboembolism, a correct antibiotic prophylaxis, the prevention of intraoperative hypothermia, prevention of volume overload, prevention of postoperative nausea and vomiting, very limited use of the nasogastric tube, early removal of the urinary catheter, multimodal analgesia to minimize opiate consumption, early postoperative mobilization and early post-operative feeding to promote rapid recovery of gastro-intestinal functions.  Other Name: ERAS (Enhanced Recovery After Surgery) protocol |
| **Study Arms** | - No Intervention: Usual care   Perioperative care for hysterectomy of benign pathologies or malignant tumors of the uterus is managed according to current hospital clinical practice.   - Experimental: ERAS protocol   Perioperative care for hysterectomy of benign pathologies or malignant tumors of the uterus is managed according to the ERAS protocol.  Intervention: Procedure: ERAS protocol |
| **Primary Outcome Measures** | **Length of stay [ Time Frame: 10 days after admission ]**  Mean length of stay calculated as difference between date of discharge and date of admission of the hospitalization for surgery, excluding patients with LOS exceeding a predefined threshold, corresponding to the 98^th^ percentile of the distribution. |
| **Secondary Outcome Measures** | **Length of stay longer than the threshold [ Time Frame: 30 days after admission ]**  Percentage of LOS longer than the threshold corresponding to the 98^th^ percentile of the distribution  **Recovery after surgery [ Time Frame: 24 hours after surgery ]**  Score of quality of recovery at 24 hours after surgery, assessed with the questionnaire Quality of Recovery (QoR-15), a 15-items instrument, with responses recorded on a 11-point Likert-type scale form 0 (worst scenario) to 10 (best scenario) and an overall score ranging from 0 (poor recovery) to 150 (excellent recovery). A visual analogue scale (VAS), ranging from 0 (worst imaginable health state) to 10 (worst imaginable health state) is also supplied as summary evaluation.  **Complications [ Time Frame: 30 days after discharge ]**  Rate of surgical and medical complication after surgery. For surgical complications: Comprehensive Complication Index  **Transfer to intensive care unit [ Time Frame: 30 days after surgery ]**  Rate of transfers to intensive care unit after surgery  **Emergency visits after discharge [ Time Frame: 30 days after discharge ]**  Rate of emergency visit in the first month after discharge  **Hospital admissions after discharge [ Time Frame: 30 days after discharge ]**  Rate of new admissions in the first month after discharge  **Reintervention [ Time Frame: 30 days after surgery ]**  Rate of reintervention in the first month after surgery, excluding planned interventions  **Patients' satisfaction [ Time Frame: 15 days after discharge ]**  Score of patients' satisfaction measured 2 weeks after discharge, assessed with the questionnaire Surgical Satisfaction Questionnaire (SSQ8) supplied by telephone. SSQ8 is a 8-items instrument, with responses recorded on a 5-point Likert-type scale from 0 (worst scenario) to 4 (best scenario) and an overall score ranging from 0 (very unsatisfied) to 32 (very satisfied).  **Healthcare costs [ Time Frame: 30 days after discharge ]**  Mean healthcare costs from pre admission visit to 30 days after discharge |
| **Recruitment Information** |  |
| **Recruitment Status** | Closed |
| **Estimated Enrollment** | 1800 |
| **Study Completion Date** | 31/05/2021 |
| **Eligibility Criteria** | **Inclusion Criteria:**   - All the hospital wards within the Piemonte Region performing hysterectomy. - All the patients receiving an elective hysterectomy for benign or malignant tumors of the uterus.   **Exclusion Criteria:**   - Hospital wards performing less than 20 expected cases per year - Emergency hysterectomy - Hysterectomy for pelvic floor disorders   High severity cases not allowing ERAS protocol implementation (i.e. American Society of Anesthesiologists score: ASA V). |
| **Ages** | 18 Years and older |
| **Listed Location Countries** | Italy |

**Statistical plan**

The sample size was estimated according to the available data on volume of hysterectomies performed in Piedmont Region in 2018. The total number of centers meeting the inclusion criteria of at least 20 hysterectomies per year was 22 with an average of approximately 88 interventions/year (around 1900 expected cases in one year). The randomization calendar includes 4-5 centers every quarter, therefore 4 periods (15 months in total, including the 3 months at baseline) are needed to complete the implementation of the ERAS protocol in all the enrolled centers. Figure 1 describes the sequence followed to randomize the clusters, the number of centers involved and the expected number of patients in the control and experimental periods. The total number of cases expected in 15 months is around 2400 patients (about 200 cases in the control period and 1200 in the experimental period). The statistical power of the study is calculated for both the main endpoint (length-of-stay), and for the dichotomous secondary endpoints, according to the sample size and the study design, applying the Hemming and Girling method with STATA software (v.13).

The power is calculated assuming that the application of the protocol entails a reduction in the average length of stay (LOS) (calculated after excluding patients with LOS exceeding a predefined threshold, corresponding to the 98^th^ percentile of the distribution) of at least 1 day (from 4.2 to 3.2), which in relation to the standard deviation (2.0) represents an effect size of about 0.5. With an alfa error (two sided) of 0.05 and an Intra Cluster Correlation (ICC) of 0.20, the total number of expected cases (2400) has a statistical power of 0.99.

The statistical power of the study is also calculated to highlight as statistically significant absolute differences of at least 10% of the secondary endpoints measurable as percentages (e.g. adherence to the ERAS protocol, complications, re-interventions, etc ...). Assuming a reference value of 0.5 (the most unfavorable from a statistical point of view), and keeping the same previous parameters, the study sample size has a statistical power of 0.82.

The mean LOS (calculated after excluding the durations greater than the threshold) will be compared between the two study periods using a random-effect linear regression model, adjusting for the time effect and the surgical technique (laparoscopic vs. open surgery). The centers will be included in the model as a random effect. For dichotomous endpoints measured as proportions (e.g. length of stay above the threshold, complications, readmissions), the effect of implementing the ERAS protocol will be estimated with logistic regression models, with centers as random effects, including in the model the same set of covariates used for the analysis of the LOS.

Planned subgroup analyses will be performed by characteristics of the centers (classified by volume of activity, degree of adherence to the ERAS protocol at the baseline and other structural characteristics) and by patients’ characteristics (age, education, ASA score, comorbidity, group of diagnosis, surgical technique and by group of centers, stratified according to the completeness of enrollment).

To take into consideration the time needed to implement the ERAS procedure, a sensitivity analysis is planned and will exclude the first month of each implementation period of the ERAS protocol. The adoption of the ERAS protocol will also be analyzed based on the time elapsed since its introduction, to evaluate the achievement curve of an acceptable and optimal level of application.

Secondary analyses will be performed to assess the adherence of the centers to the specific items of the ERAS protocol and the relationship between protocol adherence and clinical outcomes.

No interim analyses were planned, but the study was strictly monitored for data quality and safety events.

**Data collection**

The CRF is available in a dedicated area of the EPICLIN electronic platform. EPICLIN is developed and managed by the Clinical Epidemiology Unit, compliant with all the security requirements of the General Data Protection Regulation (EU regulation 2016/679). Data related to the peri-operative care are prospectively and uniformly collected in the database during the entire study. At the time of the preadmission visit, patients receive adequate information (verbal and written) on study aims and data treatment, sign an informed consent, are enrolled and their data are entered into the study database (that assigns a unique, anonymised code, to each patient). This code is also registered on the patient CRF. Clinical data collected during the hospital stay are recorded on the CRF by the local ERAS team. The post-operative recovery will be measured at 24 hours after the intervention through the QoR-15 questionnaire (available and validated in English) (16) and translated in Italian.

When the patient is discharged and the pathology report becomes available, the completed CRF and the QoR-15 questionnaire are sent to the coordinating centre for quality checks and data entry.

Patients’ satisfaction is measured through the SSQ-8 questionnaire, available and validated in English (19) (and translated in Italian), administered by trained staff to a sample of patients (or alternatively their caregivers) during a telephone interview two weeks after discharge.

At the end of the study health professionals’ satisfaction will be assessed qualitatively through questionnaires and focus groups.

Healthcare costs incurred between the first pre-admission visit and 30 days after hospital discharge, will be evaluated including the following categories of resources: pre-intervention visits, hospital stay days (including intensive care days), type of intervention, treatment of complications, re-interventions, ED access, new hospitalizations.


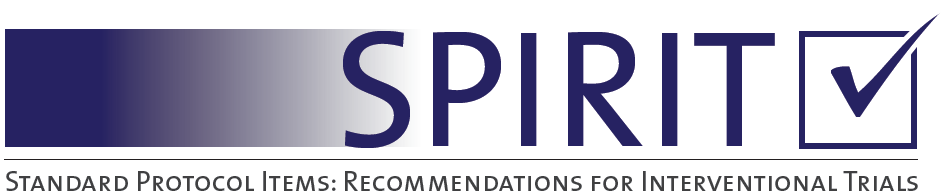


SPIRIT 2013 Checklist: Recommended items to address in a clinical trial protocol and related documents*

| Section/item | ItemNo | Description | PAG. |
| --- | --- | --- | --- |
| **Administrative information** | | |  |
| Title | 1 | Descriptive title identifying the study design, population, interventions, and, if applicable, trial acronym | 1, line 1 |
| Trial registration | 2a | Trial identifier and registry name. If not yet registered, name of intended registry | 2, line 28 |
|  | 2b | All items from the World Health Organization Trial Registration Data Set | NR |
| Protocol version | 3 | Date and version identifier | 11, line 1 |
| Funding | 4 | Sources and types of financial, material, and other support | Funding Statement of the online submission form |
| Roles and responsibilities | 5a | Names, affiliations, and roles of protocol contributors | 1, line 8 + 12, line 13 |
|  | 5b | Name and contact information for the trial sponsor | NA |
|  | 5c | Role of study sponsor and funders, if any, in study design; collection, management, analysis, and interpretation of data; writing of the report; and the decision to submit the report for publication, including whether they will have ultimate authority over any of these activities | NA |
|  | 5d | Composition, roles, and responsibilities of the coordinating centre, steering committee, endpoint adjudication committee, data management team, and other individuals or groups overseeing the trial, if applicable (see Item 21a for data monitoring committee) | 6, line 5 |
| Introduction |  |  |  |
| Background and rationale | 6a | Description of research question and justification for undertaking the trial, including summary of relevant studies (published and unpublished) examining benefits and harms for each intervention | 4, line 2 |
|  | 6b | Explanation for choice of comparators | 5, line 21 |
| Objectives | 7 | Specific objectives or hypotheses | 5, line 6 |
| Trial design | 8 | Description of trial design including type of trial (eg, parallel group, crossover, factorial, single group), allocation ratio, and framework (eg, superiority, equivalence, noninferiority, exploratory) | 5, line 21 |
| Methods: Participants, interventions, and outcomes | | |  |
| Study setting | 9 | Description of study settings (eg, community clinic, academic hospital) and list of countries where data will be collected. Reference to where list of study sites can be obtained | 5, line 28 |
| Eligibility criteria | 10 | Inclusion and exclusion criteria for participants. If applicable, eligibility criteria for study centres and individuals who will perform the interventions (eg, surgeons, psychotherapists) | 6, line 12 |
| Interventions | 11a | Interventions for each group with sufficient detail to allow replication, including how and when they will be administered | 7, line 18 |
|  | 11b | Criteria for discontinuing or modifying allocated interventions for a given trial participant (eg, drug dose change in response to harms, participant request, or improving/worsening disease) | NA |
|  | 11c | Strategies to improve adherence to intervention protocols, and any procedures for monitoring adherence (eg, drug tablet return, laboratory tests) | 8, line 5 |
|  | 11d | Relevant concomitant care and interventions that are permitted or prohibited during the trial | NA |
| Outcomes | 12 | Primary, secondary, and other outcomes, including the specific measurement variable (eg, systolic blood pressure), analysis metric (eg, change from baseline, final value, time to event), method of aggregation (eg, median, proportion), and time point for each outcome. Explanation of the clinical relevance of chosen efficacy and harm outcomes is strongly recommended | 8, line 24 |
| Participant timeline | 13 | Time schedule of enrolment, interventions (including any run-ins and washouts), assessments, and visits for participants. A schematic diagram is highly recommended (see Figure) | 7, line 1;  Figure 1 |
| Sample size | 14 | Estimated number of participants needed to achieve study objectives and how it was determined, including clinical and statistical assumptions supporting any sample size calculations | 10, line 5 |
| Recruitment | 15 | Strategies for achieving adequate participant enrolment to reach target sample size | NA |
| **Methods: Assignment of interventions (for controlled trials)** | | |  |
| Allocation: |  |  |  |
| Sequence generation | 16a | Method of generating the allocation sequence (eg, computer-generated random numbers), and list of any factors for stratification. To reduce predictability of a random sequence, details of any planned restriction (eg, blocking) should be provided in a separate document that is unavailable to those who enrol participants or assign interventions | 7, line 1 |
| Allocation concealment mechanism | 16b | Mechanism of implementing the allocation sequence (eg, central telephone; sequentially numbered, opaque, sealed envelopes), describing any steps to conceal the sequence until interventions are assigned | NA |
| Implementation | 16c | Who will generate the allocation sequence, who will enrol participants, and who will assign participants to interventions | NA |
| Blinding (masking) | 17a | Who will be blinded after assignment to interventions (eg, trial participants, care providers, outcome assessors, data analysts), and how | NA |
|  | 17b | If blinded, circumstances under which unblinding is permissible, and procedure for revealing a participant’s allocated intervention during the trial | NA |
| **Methods: Data collection, management, and analysis** | | |  |
| Data collection methods | 18a | Plans for assessment and collection of outcome, baseline, and other trial data, including any related processes to promote data quality (eg, duplicate measurements, training of assessors) and a description of study instruments (eg, questionnaires, laboratory tests) along with their reliability and validity, if known. Reference to where data collection forms can be found, if not in the protocol | 9, line 14 |
|  | 18b | Plans to promote participant retention and complete follow-up, including list of any outcome data to be collected for participants who discontinue or deviate from intervention protocols | NA |
| Data management | 19 | Plans for data entry, coding, security, and storage, including any related processes to promote data quality (eg, double data entry; range checks for data values). Reference to where details of data management procedures can be found, if not in the protocol | NR |
| Statistical methods | 20a | Statistical methods for analysing primary and secondary outcomes. Reference to where other details of the statistical analysis plan can be found, if not in the protocol | 10, line 3 |
|  | 20b | Methods for any additional analyses (eg, subgroup and adjusted analyses) | 10, line 17 |
|  | 20c | Definition of analysis population relating to protocol non-adherence (eg, as randomised analysis), and any statistical methods to handle missing data (eg, multiple imputation) | Supplemental materials (see section “Statistical plan”)  Missing data NR |
| **Methods: Monitoring** | | |  |
| Data monitoring | 21a | Composition of data monitoring committee (DMC); summary of its role and reporting structure; statement of whether it is independent from the sponsor and competing interests; and reference to where further details about its charter can be found, if not in the protocol. Alternatively, an explanation of why a DMC is not needed | NA |
|  | 21b | Description of any interim analyses and stopping guidelines, including who will have access to these interim results and make the final decision to terminate the trial | NA |
| Harms | 22 | Plans for collecting, assessing, reporting, and managing solicited and spontaneously reported adverse events and other unintended effects of trial interventions or trial conduct | NA |
| Auditing | 23 | Frequency and procedures for auditing trial conduct, if any, and whether the process will be independent from investigators and the sponsor | NR |
| Ethics and dissemination | | |  |
| Research ethics approval | 24 | Plans for seeking research ethics committee/institutional review board (REC/IRB) approval | 10, line 20 |
| Protocol amendments | 25 | Plans for communicating important protocol modifications (eg, changes to eligibility criteria, outcomes, analyses) to relevant parties (eg, investigators, REC/IRBs, trial participants, trial registries, journals, regulators) | NR |
| Consent or assent | 26a | Who will obtain informed consent or assent from potential trial participants or authorised surrogates, and how (see Item 32) | Supplemental materials (see section “Data collection”) |
|  | 26b | Additional consent provisions for collection and use of participant data and biological specimens in ancillary studies, if applicable | NA |
| Confidentiality | 27 | How personal information about potential and enrolled participants will be collected, shared, and maintained in order to protect confidentiality before, during, and after the trial | 9, line 15 |
| Declaration of interests | 28 | Financial and other competing interests for principal investigators for the overall trial and each study site | 13, line 14 |
| Access to data | 29 | Statement of who will have access to the final trial dataset, and disclosure of contractual agreements that limit such access for investigators | NR |
| Ancillary and post-trial care | 30 | Provisions, if any, for ancillary and post-trial care, and for compensation to those who suffer harm from trial participation | NA |
| Dissemination policy | 31a | Plans for investigators and sponsor to communicate trial results to participants, healthcare professionals, the public, and other relevant groups (eg, via publication, reporting in results databases, or other data sharing arrangements), including any publication restrictions | 10, line 28 |
|  | 31b | Authorship eligibility guidelines and any intended use of professional writers | NA |
|  | 31c | Plans, if any, for granting public access to the full protocol, participant-level dataset, and statistical code | NR |
| Appendices |  |  |  |
| Informed consent materials | 32 | Model consent form and other related documentation given to participants and authorised surrogates | NR |
| Biological specimens | 33 | Plans for collection, laboratory evaluation, and storage of biological specimens for genetic or molecular analysis in the current trial and for future use in ancillary studies, if applicable | NA |

*It is strongly recommended that this checklist be read in conjunction with the SPIRIT 2013 Explanation & Elaboration for important clarification on the items. Amendments to the protocol should be tracked and dated. The SPIRIT checklist is copyrighted by the SPIRIT Group under the Creative Commons “[Attribution-NonCommercial-NoDerivs 3.0 Unported](http://www.creativecommons.org/licenses/by-nc-nd/3.0/)” license.

LEGEND: NA= not applicable; NR= not reported.

**RECOvER** Checklist for reporting of enhanced recovery research

| Section | ItemNo | Recommendation | PAG. |
| --- | --- | --- | --- |
| *Title* |  |  |  |
| Title | 1 | Indicate that this is an enhanced recovery study in the title | 1, line 1 |
| *Introduction* |  |  |  |
| Background | 2 | Explain the area of uncertainty that the study seeks to address | 4, line 2 |
| Guidelines | 3 | If a published set of enhanced recovery guidelines exists for this procedure, include a reference to the guidelines | 4, line 5 |
| Outcomes | 4 | Define the primary outcome and any key prespecified secondary outcomes for the study | 8, line 24 |
| *Methods* |  |  |  |
| IRB approval | 5 | Give the Institutional Review Board/Ethics Committee name and approval number. If permission was not required, reasons should be stated | 10, line 21 |
| Study design | 6 | Indicate what type of study is presented (randomized controlled trial, cohort, cross-sectional, etc.) The individual guidelines for the type of study should be followed (e.g., CONSORT for randomized controlled trial, STROBE for cohort studies, etc.) | 5, line 21  SPIRIT in Supplemental materials |
| Setting | 7 | Describe whether this is a single or multicenter study, the type of practice (academic vs. community, tertiary vs. primary), and the providers (limited group or all providers on a service) | 5, line 21 |
| Timing | 8 | Describe periods of recruitment, time points at which outcomes assessed, and follow-up | NR |
| Partecipants | 9 | Define study inclusion and exclusion criteria | 6, line 11 |
| Enhanced recovery protocol | 10 | Describe when the enhanced recovery protocol was implemented relative to the study period | 7, line 6 |
|  | 11 | Provide a flow diagram or table through the continuum of care detailing the enhanced recovery protocol including the following elements:  (a) Preadmission patient education regarding the protocol  (b) Preadmission screening and optimization as indicated for nutritional deficiency, frailty, anemia, HbA1c, tobacco cessation, and ethanol use  (c) Fasting and carbohydrate loading guidelines (d) Preemptive analgesia (dose, route, timing) (e) Anti-emetic prophylaxis (dose, route, timing) (f) Intraoperative fluid management strategy  (g) Types, doses, and routes of anesthetics administered (h) Patient warming strategy (i) Management of postoperative fluids (j) Postoperative analgesia and anti-emetic plans  (k) Plan for opioid minimization (l) Drain and line management (m) Early mobilization strategy (n) Postoperative diet and bowel regimen management (o) Criteria for discharge  (p) Tracking of post-discharge outcomes | NR |
| Enhanced recovery auditing | 12 | Describe the audit system for compliance with the enhanced recovery protocol and how compliance data are measured | 8, line 3 |
| Outcomes | 13 | (a) Explain the criteria for assessing primary and secondary outcomes (b) Distinguish among clinical, functional, administrative, and quality of life outcome measures | 8, line 24 |
| PROs | 14 | If patient questionnaires are used, provide references to validation of these study instruments | 9, line 8 |
| *Results* |  |  |  |
| Patient population | 15 | Use a flow diagram to explain the derivation of the study population (a) Provide a Table I with the key demographic and clinical features of the study population (b) Indicate number of participants with missing data for each variable of interest | NA |
| Enhanced recovery compliance | 16 | Provide a Table II with average compliance for each enhanced recovery protocol element and present a comparison of the variation in enhanced recovery compliance among the study groups | NA |
| Correlations | 17 | Perform logistic regression to correlate the change in primary outcome with the study intervention | NA |
| *Discussion* |  |  |  |
| Context | 18 | Explain what the study adds to the body of knowledge regarding the study intervention within the context of enhanced recovery after surgery care | 11, line 8 |
| Limitations | 19 | Discuss the limitations of the study and how these might temper the findings | 11, line 20 |
| *Other information* |  |  |  |
| Funding | 20 | Document all sources of funding and potential conflicts of interest for the study authors | 13, line 14 and Funding Statement of the online submission form |
| LEGEND: NA= not applicable; NR= not reported. | | | |
