# Supplementary material for: Implementation of the ERAS (Enhanced Recovery After Surgery) protocol for hysterectomy in the Piedmont Region with an audit&feedback approach: Study protocol for a stepped wedge cluster randomized controlled trial. A study of the EASY-NET project
Source: PLoS One. 2022 May 27;17(5):e0268655. doi: 10.1371/journal.pone.0268655 (PMC9140274; doi:10.1371/journal.pone.0268655)
Supplement: S2 File — (PDF) [file pone.0268655.s002.pdf]

**Title**	**Randomized controlled cluster study - stepped wedge - on the implementation of the ERAS protocol (Enhanced Recovery After Surgery) in the perioperative management of patients undergoing hysterectomy for benign gynecological pathology or for neoplasia of the neck-body of the uterus in the Piedmont Region. A study from the EASY-NET project.**
**Short title**	**ERAS Hysterectomy Project**
**Version**	2.0 (January 2020)
**Promoter**	ASO Santa Croce and Carle of Cuneo<br>SC Obstetrics and Gynecology
**Funding**	The study is included among the activities of the Network Program of Health Research Finalized of the Ministry of Health Year 2016 (NET-2016- 02364191) financed by the Ministry of Health and the Piedmont Region (EASY-NET).

**Principal investigators**	Marco Camanni, MD<br>Martini Hospital - ASL City of Turin, Turin<br>Head of Gynecological Surgery POIS<br><br>Andrea Puppo, MD<br>S. Croce and Carle Hospital, Cuneo (promoter center and study coordinator)<br>Director of SC Gynecology and Obstetrics<br><br>Giovannino Ciccone, MD PhD<br>Unit of Clinical and Evaluative Epidemiology, "City of Health and Science" of Turin and CPO Piedmont, Turin<br>PI of WP3 of the network program of RSF2016 (EASY-NET)
**Scientific Committee**	Elena Maria Delpiano, MD<br>SC Obstetrics and Gynecology<br>Martini Hospital - ASL City of Turin, Turin<br><br>Laura Ceretto Giannone, MD<br>Anesthesia and Service<br>Gradenigo Humanitas Hospital, Turin<br><br>Eva Pagano, MSc<br>Unit of Clinical and Evaluative Epidemiology, "City of Health and Science" of Turin and CPO Piedmont, Turin<br>coPI of WP3 of the network program of RSF2016 (EASY-NET)<br><br>Elisa Piovano, MD PhD<br>SC Obstetrics and Gynecology<br>Regina Montis Regalis Hospital, Mondovì (CN)<br><br>Andrea Scoletta, MD<br>SC Obstetrics and Gynecology<br>Santa Croce Hospital, Moncalieri (TO)<br><br>Paolo Zola, MD<br>City of Health and Science of Turin, S. Anna Hospital, Turin<br>Coordinator of the study group Gynecological Tumors - Piedmont Oncological Network Valle d'Aosta<br><br>Monica Rolfo<br>Head of Nursing Structure Humanitas Cellini and Clinica Fornaca Sessant, Turin<br>National Nursing Contact POIS for the four-year period 2016/2019<br><br>Anna Maria Orlando, MA<br>Health Department,<br>Specialized Assistance Sector and Hospital,<br>Piedmont Region<br><br>Oscar Bertetto; MD, PhD<br>Director of the Inter-company Interregional Oncology Network Department of Piedmont and Valle d'Aosta, AOU City of Health and Science of Turin.

Index		
	Background	Pag. 4
	Hypothesis	5
	Main objective	6
	Secondary objectives	6
	Study design	6
	Inclusion criteria	6
	Exclusion criteria	7
	Methods of stratification and randomization of the centers	7
	Standard peri-operative management	7
	Experimental management (ERAS)	7
	Endpoint	8
	Methods and tools for data collection and feedback	8
	Statistical considerations on numerosity and power of the study	9
	Analysis Statistics	10
	Bibliography	11
**Annexes**	1) ERAS protocol for the perioperative management of patients undergoing hysterectomy for benign or malignant gynecological pathology	14
	2) Data collection form (Case Report Form - CRF)	19
	3) QoR-15 questionnaire to assess the quality of post-operative recovery	20
	4) SSQ-8 questionnaire to assess patient satisfaction after discharge	21
	5) Patient information leaflet	22
	6) MUST score	23
	7) ESPEN 2018 consent document	24
	8) Sample form for monitoring the intake of preoperative nutritional supplement	25
	9) Example of patient diary for the post surgery monitoring of mobilization and food and beverage intake	26

**Background**	The ERAS (Enhanced Recovery After Surgery) protocol refers to a certain number of perioperative interventions which aim to alleviate surgical stress, optimize pain management and maintain the patient's normal physiology as much as possible (1-2).<br>The main objectives of ERAS are:<br>● optimize perioperative management using procedures based on scientific evidence<br>● favor a better recovery of autonomy in the post-operative period<br>● favor a decrease in hospitalization times<br>● increase the level of patient satisfaction regarding the care received<br>● reduce the incidence of complications, hospital readmissions and costs (3-5).<br><br>After the publication of numerous studies in the field of general surgery (6-7), the ERAS protocols are spreading, gaining increasing interest also in the gynecological field, thanks to the publication in 2016 of specific guidelines by the ERAS Society (8- 9).<br><br>In the gynecological field, the ERAS strategy provides for an accurate counselling with the patient in the preoperative phase aimed at stopping smoking and possibly hormonal therapies, the reduction of preoperative fasting, the omission of bowel preparation, the prophylaxis of thromboembolism, a correct antibiotic prophylaxis, prevention of intraoperative hypothermia, perioperative euvolemia, prevention of postoperative nausea and vomiting, very limited use of the nasogastric tube, early removal of the urinary catheter, multimodal analgesia to minimize opioid consumption, early postoperative mobilization and early post-operative nutrition, to promote a rapid recovery of gastrointestinal functions (8-9, 21).<br><br>A recent publication from the Royal College of Obstetricians and Gynecologists (10) examines the main elements of ERAS management and concludes that ERAS programs offer safe, high-quality perioperative care, and should become standard practice for all women undergoing elective gynecological surgery.<br>However, to date, most of the published studies on the benefits of an ERAS program over standard care in gynecological surgery are cohort studies and often analyze the effect of individual ERAS management specific procedures; only rarely is the “ERAS protocol” tested as a whole within a randomized trial (11). Scientific evidence regarding ERAS in gynecological cancer surgery is even rarer (12-14).<br>All this is reflected in a slow diffusion of the ERAS principles in clinical practice.<br>An international survey was recently conducted (Scandinavian countries, Italy, Austria) on the perioperative management of patients treated for ovarian cancer (data to be published). The survey clearly demonstrated that the ERAS principles have been implemented very unevenly at European level, with Italy particularly late in updating the perioperative protocols. The survey also demonstrated that the centralization of the management of complex pathologies, such as ovarian cancer, favors an updated and evidence-based operative management.

	A similar survey was also conducted in the Piedmont Region, to investigate how much of the ERAS principles had already been incorporated in the management of surgical gynecological pathologies. Although a rather “traditional” perioperative management emerged, all respondents (22/22) reported a strong interest in the ERAS approach and participation in shared initiatives aimed at implementing these principles.<br><br>A formal program for implementing the ERAS protocol requires three elements (13-15):<br>i) an updated and shared ERAS operating protocol<br>ii) a team working to train operators and to increase compliance with the protocol<br>iii) an audit and feedback system (with dedicated database) to verify compliance with the protocol and to monitor clinical outcomes.<br><br>In Italy, the dissemination of the ERAS strategy is the goal of the Perioperative Italian Society - POIS ERAS Italian Chapter (http://perioperativeitaliansociety.org) whose mission is to promote the minimally invasive surgical procedure and improve the patient's quality of life in the perioperative period.<br>The Society has activated a network of over 70 Italian hospitals and has developed a database for colorectal surgery which has allowed the recent publication of multicentre studies (16-18).<br>In the gynecological field, the ERAS-POIS protocol recently drawn up and revised, will be the reference of the study in question (Annex 1).<br><br>The hypothesis of testing the application of the ERAS protocol for some selected interventions (in particular for colorectal surgery and hysterectomy) and of evaluating its impact in terms of improving efficiency and safety was considered a regional priority, such as to include it in the more general network project on the evaluation of the effectiveness of audit and feedback interventions (Network project - Finalized Health Research 2016). In this network project (NET-2016-02364191), which involves 7 regions at national level, with the coordination of the Lazio Region, the Piedmont Region is responsible for Work Package 3 (WP3) which involves the use of a controlled and randomized cluster to estimate the effect on the entire hospital system of interventions to improve the quality of care in the oncology field, with an intensive audit and feedback approach based on data collected in dedicated databases, centrally managed, compared to traditional approaches of audits based on local initiatives or with central indicators that use only current data.
**Hypothesis**	This study was designed on the basis of two main hypotheses:<br>a) the ERAS protocol has a high probability, based on the available evidence, of introducing procedures in clinical practice with a favorable balance between benefits and risks (both for patients and for staff);<br>b) the simple dissemination of the protocol in selected hospitals favorably predisposed to change would have a limited impact on the overall quality of interventions on a regional scale, with a consequent accentuation of the heterogeneity of services between centers and a reduction in equity among patients.

	Taking into account the available evidence and the opportunities provided by the regional context, the Department of Health of the Piedmont Region considered the implementation of the ERAS protocol in all regional hospitals, as part of a research project, a high priority objective.<br>On the basis of these premises, was designed a research/intervention project which should, in a period of about 2 years, facilitate a correct knowledge and systematic adoption of the ERAS protocol on a regional scale by all the centers that perform gynecological interventions. In order to accurately estimate the real impact of the protocol, a randomized cluster study was designed (where the clusters are represented by the gynecology departments of regional hospitals) with progressive adoption of the protocol by groups of departments according to a calendar determined in random mode (defined as "stepped wedge") which at the end of the study will have involved all the clusters. At the end of the study, each cluster will have a period of activity with usual procedures ("control period") and one subsequent to the introduction of the protocol ("experimental period") similar to the designs with cross-over, but with only one transition (from control to experimental).<br>We hypothesize that the adoption of the protocol determines a reduction in the length of hospitalization, complications, health costs and improves functional recovery and patient satisfaction.
**Main objective**	To reduce the duration of total hospitalization in the experimental period compared to the control period through the implementation of the ERAS standardized perioperative protocol for the management of patients who are candidates for hysterectomy (ERAS-POIS protocol).
**Secondary objectives**	Verify that in the transition from the control period to the experimental period there is a reduction in the frequency of:<br>● post-surgical complications<br>● transfers to the intensive care unit<br>● hospital stays longer than the threshold<br>● reoperations<br>● access to the emergency room<br>● readmissions to the hospital<br>Evaluate compliance with the ERAS protocol, overall and for the individual aspects / procedures.<br>Compare between the two periods:<br>● the use of the laparoscopic technique.<br>● the quality of the post-operative recovery<br>● the perceived satisfaction of the patients<br>● the economic impact<br>In the experimental period analyze:<br>● the organizational and structural factors of the departments associated with compliance with the ERAS protocol<br>● the relationship between compliance with the ERAS protocol and patient outcomes<br>● operator satisfaction.
**Study Design**	A multicenter randomized controlled cluster study, with *stepped wedge* design, comparing between the standard perioperative management and the management according to the ERAS protocol.
**Inclusion criteria**	All gynecological surgery departments of regional hospitals that perform hysterectomy.

	All patients who during the study are hospitalized for elective hysterectomy surgery for benign gynecological pathology or for neoplasia of the neck or body of the uterus.
**Exclusion criteria**	Departments with a caseload of less than 20 patients per year (less than 20 hysterectomy operations per year).<br>Patient candidates for hysterectomy surgery for pelvic floor disease.<br>Patients undergoing an emergency hysterectomy.<br>Particular conditions of clinical complexity or seriousness, to be documented at the time of admission, which represent contraindications to the application of the ERAS protocol.<br>Patients with socio-assistance discomfort, to be documented at the time of admission, who do not allow an adequate standard of personal autonomy or post-operative home care (conditions of severe dementia, physical dysfunction, state of indigence) will be included and managed in accordance with the provisions of the study, except for the discharge decision which will have to be evaluated in relation to the specific problems (information provided in the database).
**Methods of stratification and randomization of the centers**	On the basis of a regional planning choice, all the centers accredited to carry out gynecological surgery in the Piedmont Region that meet the specific inclusion criteria of the study are included in the study.<br>Before the start of the study, all centers will be contacted by the coordination group to assess the level of knowledge of the ERAS protocol for gynecological surgery, the predisposition to adopt it (with registration of the main existing barriers) or any previous adoption.<br>Centers found to have fully adopted the protocol prior to the start of the study will be excluded from randomization and included in an observational group.<br>All other centers will be sorted according to the volume of hysterectomies performed during 2017 and divided into 4 layers (with an equal number of departments per layer). Groups of 4 centers (one per layer) will then be randomly extracted and the groups thus formed will be ordered according to a random sequence of protocol activation periods. In this way, a number of interventions and a homogeneous composition of the groups are guaranteed for each activation period. All randomization procedures will be performed after having anonymized the centers. The activation date of the protocol and of the training event will finally be communicated to each center with a notice of about 3 months (time necessary to allow staff training and the preparation of local organizational aspects). All the randomization procedures will be performed centrally by the Clinical and Evaluative Epidemiology Department of the CPO - Piedmont.
**Standard peri-operative management**	Before the protocol is activated, each center will continue to manage the patients' perioperative according to the usual protocols, possibly without introducing changes.
**Experimental management (ERAS)**	For each group of about 4-6 centers in the quarter preceding the protocol activation date, an in-depth training will be carried out on the ERAS principles and the new ERAS-POIS protocol (annex 1). The training will consist of a one-day course run by expert POIS trainers.<br>Each participating center must identify, in addition to the director of the structure and the nursing coordinator, a professional reference for each professional figure involved in the project (gynecologist, anesthetist, nurse, dietician, medical director). The regional “TEAM ERAS” will participate in the training and will subsequently make itself available for

	further information and to provide support for the implementation of the protocol and training at the local level.
**Endpoint**	Primary:<br>● mean length of stay, calculated excluding durations above a predefined threshold of 12 days (corresponding to the 98th percentile of the distribution of hospital stays in 2018)<br>Secondary:<br>● incidence of general and post-surgical complications in the post-operative period, defined according to the Clavien-Dindo classification<br>● transfers to the intensive care unit in the post-operative period<br>● percentage of hospitalizations lasting longer than the threshold (> 12 days)<br>● percentage of re-operations (within the same hospitalization or in any case in the 30 days following the intervention)<br>● percentage of accesses to the emergency room within 30 days of surgery (regardless of the reason and possible hospitalization)<br>● percentage of readmissions to hospital within 30 days of surgery (regardless of the reason)<br>● percentage of operations performed with laparoscopic technique<br>● quality score of post-operative recovery (measured by the QoR-15 questionnaire [19] about 24 hours after the surgery<br>● score of satisfaction perception of patients measured with the SSQ-8 questionnaire [20] by telephone interview two weeks after discharge (only for a sample of patients who agree to be interviewed)<br>● assessment of operator satisfaction, qualitatively detected through focus groups, at end of the trial<br>● average care costs, calculated from pre-hospitalization up to 30 days after surgery.
**Methods and tools for data collection and feedback**	Before the start of the study, all departments will be enabled to enter the data of patients undergoing hysterectomy included in the study in a dedicated area on an electronic platform (developed and managed by the Clinical Epidemiology Department and Evaluation - CPO of the CSS of Turin). In the database will be prospectively recorded and after reversible anonymization the data relating to the management of the peri-operative period of all patients undergoing hysterectomy who will have agreed to make their data available for the purposes of the study by signing a consent form, after having received adequate written and oral information during the pre-admission period. The data collection form (Case Report Form - CRF) is shown in Annex 2.<br>The members of the ERAS TEAM of each center will have access to this platform through individual IDs and passwords and will be able to view and modify only the data relating to their patients. The database will be developed in compliance with all the security requirements provided by the GDPR (EU law 2016/679) in force since 25/5/2018 and subsequent amendments.<br>Therefore, after the start of the study (which will take place simultaneously in all centers), each center will register on the web platform the data relating to the standard management of its patients who are candidates for hysterectomy. Subsequently, based on the ERAS protocol activation calendar in each center, the department's team will also have access to the monitoring section of the area in which graphs (with simultaneous updating of data) will be available showing

	the main endpoints of the study, information on the general progress of the protocol at the regional level and of the specific center. This form of feedback, based on process indicators (adherence of clinical practice to the procedures of the ERAS protocol) and outcome (length of hospitalization, secondary endpoints) should allow each department to promptly identify critical issues in order to address them and introduce corrective actions.<br>Post-operative recovery will be measured approximately 24 hours after surgery through the QoR-15 questionnaire (available and validated in English). The Italian version was obtained through a process of translation and retro translation ("forward / backward translation") and subsequent validation on a first sample of patients (annex 3). To this end, patients will also be given a visual analog scale (VAS), with a scale between “poor recovery” and “excellent recovery”, as a synthetic measure of the quality of recovery.<br>The satisfaction perceived by the patients will be measured through the SSQ-8 questionnaire (annex n.4), administered by telephone to a sample of patients or their caregivers two weeks after discharge, by personnel experienced in administering health questionnaires. Since the questionnaire, validated and available in English, is not available in Italian, the Italian version was obtained through a process of translation and retro translation ("forward / backward translation") and subsequent validation on a first sample of patients, through the correlation with complications, reoperations, accesses to the ED and readmissions.<br>The evaluation of the operators' satisfaction will be carried out through questionnaires and, in a qualitative way, through the conduct of focus groups, in the final phase of the study, involving separately the different groups of professionals (nurses, gynecologists, anesthetists). The focus groups will be conducted by experienced staff.<br>The costs of care will be assessed by including the following categories of resources: pre-intervention assessments, days of hospitalization (including days in intensive care), type of intervention, treatment of complications, re-operations, access to the ED, new hospitalizations.<br>For the assessment of the organizational and structural factors of the departments associated with compliance with the ERAS protocol, it is intended to administer a short questionnaire to the participating centers, during the conduct of the training courses, in order to collect some contextual information (size of the department, organization, knowledge and preparation for the ERAS protocol, critical issues).
**Statistical considerations on the size and power of the study**	Taking into account that in 2018 the 22 centers operating in the region (which have not yet adopted ERAS) performed a total of about 1900 scheduled hysterectomies, with an average of 22 operations per quarter for each center, each group of 4-5 centers should perform about 90 surgeries per quarter. With an activation calendar that includes a group of 4-5 centers each quarter, after an initial quarter, 4 quarters (15 months in total) are required to complete the implementation of the ERAS protocol on all centers in the region.<br>The scheme of the following table shows a possible sequence of activation of the clusters during the study with the number of wards and patients for the control and experimental period. With this hypothesis of expected size (n = 2400, of which 1200 interventions in the control period and 1200 in the experimental period) and design, the statistical power of the study was calculated both for the main endpoint (length of

	hospital stay) and for safety endpoints (frequency of complications, reoperations).<br><br>To calculate the statistical power of the study, the Hemming and Girling method was applied using the STATA software (v. 13).<br>The power was calculated assuming that the application of the protocol involves a reduction in the average length of stay of at least one day (corresponding to an effect size of about 0.5) to be defined as effective.<br>The parameters used for the calculation are:<br>Mean hospital stay (standard): 4.2 days (SD: 2.0)<br>Mean hospital stay (experimental): <= 3.2 days (SD 2.0)<br>Alpha error (two-sided): 0.05<br>Correlation coefficient within cluster (ICC): 0.10<br>Average number of clusters: 22<br>Number of randomized clusters per step: 5<br>Number of steps (excluding baseline): 4<br><br>Total number of the study: 2400<br>Statistical power: 0.99<br><br>The statistical power of the study was also calculated to show statistically significant absolute differences of at least 10% of secondary endpoints measurable as percentages (eg adherence to the items of the ERAS protocol, complications, re-operations, etc…). Assuming a reference value of 0.5 (the most unfavorable value from a statistical point of view), and keeping all the previous parameters, the study has a power of 0.82.

months	0	3	6	9	12	Totale
Step 4	120	120	120	120	120	
Step 3	120	120	120	120	120	
Step 2	120	120	120	120	120	
Step 1	120	120	120	120	120	
Number per period:						
Patients (control)	480	360	240	120	0	1200
Patients (experimental)	0	120	240	360	480	1200

**Statistical Analysis**	The average hospital stays (calculated excluding the duration greater than the threshold) will be compared between the periods with and without ERAS protocol implementation using random effects linear regression models. In the model, hospitalization will be treated as a dependent variable while, among the independent ones, a dichotomous variable will indicate for each center the implementation of the protocol in the reference period of hospitalization. The effect of the implementation of the ERAS protocol on the average hospital stay will be adjusted by including in the model the variables time (identified by the foreseen steps) and the surgical technique (LPT, LPS). The center will be included in the pattern as a random effect. For dichotomous endpoints measured as proportions (e.g. lengths of stay above the threshold, complications, hospital readmissions) the effect of implementing the ERAS protocol will be estimated with random effects logistic regression models (center), using an indicator of 'event (0, 1) as

	a dependent variable and including in the model the same set of covariates used for the analysis of the length of stay.<br>The main analyzes will also be stratified by characteristics of the centers (classified by volume of activity, by degree of adherence to the ERAS protocol at baseline and by other structural characteristics) and by patients (age groups, diagnosis, surgical technique used). Centers with high adherence to the ERAS protocol at baseline will subsequently be excluded from the main analysis.<br>In order to reduce the risk of bias due to a selected inclusion of patients in the study by the centers (selection bias, assessed on the basis of the percentage of cases entered on the total of discharge forms of the same enrollment period), analyzes will be stratified for completeness of enrollment (with the possibility of exclusion of centers with greater incompleteness).<br>To take into account the adaptation period in each center, a sensitivity analysis is planned that will exclude the first month of each ERAS protocol activation period from the comparison.<br>The impact of the ERAS protocol will also be analyzed as a function of the time elapsed since its introduction to evaluate the curve for reaching an acceptable and optimal level of application.<br>As a secondary analysis, the main endpoint of the study on the entire regional case history will be assessed through the hospital discharge forms of the Piedmont region, selecting patients with the study inclusion criteria.<br>Furthermore, it is planned to evaluate the trend over time of the average length of hospital stay detectable through the hospital discharge forms in the 5 years preceding the activation of the ERAS protocol and in the following year, through an interrupted time series design.
**Bibliography**	1) Kehlet H and Wilmore DW: Evidence-based surgical care and the evolution of fast-track surgery. Ann Surg 248 (2): 189-198, 2008<br>2) Ansari D, Gianotti L, Schroder J and Andersson R: Fast-track surgery: procedure-specific aspects and future direction. Langenbecks Arch Surg 398 (1): 29-37, 2013.<br>3) O. Ljungqvist, M. Scott, KC Fearon, Enhanced recovery after surgery: a review, JAMA Surg. 152 (3) (2017 Mar 1) 292–298<br>4) Visioni, R. Shah, E. Gabriel, K. Attwood, M. Kukar, S. Nurkin, Enhanced recovery after surgery for noncolorectal surgery ?: A systematic review and meta-analysis of major abdominal surgery, Ann. Surg. (2017) (Epub ahead of print).<br>5) O. Ljungqvist, NX Thanh, G. Nelson, ERAS - value based surgery, J. Surg. Oncol. (2017 Sep 5) (Epub ahead of print).<br>6) Spanjersberg WR, Reurings J, Keus F and van Laarhoven CJ: Fast track surgery versus conventional recovery strategies for colorectal surgery. Cochrane Database Syst Rev 2, 2011.<br>7) Zhuang CL, Ye XZ, Zhang XD, Chen BC and Yu Z: Enhanced recovery after surgery programs versus traditional care for colorectal surgery: a meta-analysis of randomized controlled trials. Dis Colon Rectum 56 (5): 667-678, 2013.<br>8) Nelson G, Altman AD, Nick A, Meyer LA, Ramirez PT, Achtari C, Antrobus J, Huang J, Scott M, Wijk L, Acheson N, Ljungqvist O, Dowdy SC. Guidelines for pre- and intra-operative care in gynecologic / oncology surgery: Enhanced Recovery After Surgery

(ERAS) Society recommendations - Part I. Gynecol Oncol. 2016 Feb; 140 (2): 313-22.

9) Nelson G, Altman AD, Nick A, Meyer LA, Ramirez PT, Achtari C, Antrobus J, Huang J, Scott M, Wijk L, Acheson N, Ljungqvist O, Dowdy SC. Guidelines for postoperative care in gynecologic / oncology surgery: Enhanced Recovery After Surgery (ERAS) Society recommendations - Part II. Gynecol Oncol. 2016 Feb; 140 (2): 323-32.
10) Scientific Impact Paper N ° 36: Enhanced Recovery in Gynecology, Royal College of Obstetricians and Gynecologists, February 2013 (http://www.rcog.org.uk/files/ rcog-corp / 8.2.13SIP36.pdf).
11) Nelson G, Ramirez PT, Ljungqvist O, Dowdy SC. Enhanced Recovery Program and Length of Stay After Laparotomy on a Gynecologic Oncology Service: A Randomize Controlled Trial. Obstet Gynecol. 2017 Jun; 129 (6): 1139.
12) Lindemann K, Kok PS, Stockler M, Jaaback K, Brand A. Enhanced Recovery AftermSurgery for Advanced Ovarian Cancer: A Systematic Review of Interventions Trialed. Int J Gynecol Cancer. 2017 Jul; 27 (6): 1274-1282.
13) E.Kalogera, JN Bakkum-Gamez, CJ Jankowski, E. Trabuco, JK Lovely, S. Dhanorker, PL Grubbs, AL Weaver, LR Haas, BJ Borah, AA Bursiek, MT Walsh, WA Cliby, SC Dowdy, Enhanced recovery in gynecologic surgery, Obstet. Gynecol. 122 (2013) 319–328.
14) E. Miralpeix, AM Nick, LA Meyer, J. Cata, J. Lasala, GE Mena, V. Gottumukkala, M. Iniesta-Donate, G. Salvo, PT Ramirez, A call for new standard of care in perioperative gynecologic oncology practice: Impact of enhanced recovery after surgery (ERAS) programs, Gynecol. Oncol. 141 (2) (2016) 371–378.
15) G. Nelson, AD Altman. Enhanced recovery after surgery interactive audit system for gynecologic /oncology surgery – importance of measuring protocol element compliance (you don't know what you don't measure!), Gynecol. Oncol. 145 (Suppl. 1) (2017) 191.
16) Braga M, Beretta L, Pecorelli N, Maspero M, Casiraghi U, Borghi F, Pellegrino L, Bona S, Monzani R, Ferrari G, Radrizzani D, Iuliani R, Bima C, Scatizzi M, Missana G, Guicciardi MA, Muratore A, Crespi M, Bouzari H, Ceretti AP, Ficari F; PeriOperative Italian Society Group. Enhanced recovery pathway in elderly patients undergoing colorectal surgery: is there an effect of increasing ages? Results from the perioperative Italian Society Registry. Updates Surg. 2017 Jun
17) Braga M, Borghi F, Scatizzi M, Missana G, Guicciardi MA, Bona S, Ficari F, Maspero M, Pecorelli N; PeriOperative Italian Society. Impact of laparoscopy on adherence to an enhanced recovery pathway and readiness for discharge in elective colorectal surgery: Results from the PeriOperative Italian Society registry. Surg Endosc. 2017 Nov;31(11):4393-4399.
18) Braga M, Pecorelli N, Scatizzi M, Borghi F, Missana G, Radrizzani D; PeriOperative Italian Society. Enhanced Recovery Program in High-Risk Patients Undergoing Colorectal Surgery: Results from the PeriOperative Italian Society Registry. World J Surg. 2017 Mar;41(3):860-867.

	19) Stark PA, Myles PS, Burke JA. Development and psychometric evaluation of a postoperative quality of recovery score: the QoR-15. Anesthesiology. 2013 Jun;118(6):1332-40.<br>20) Murphy M, Sternschuss G, Haff R, van Raalte H, Saltz S, Lucente V. Quality of life and surgical satisfaction after vaginal reconstructive vs obliterative surgery for the treatment of advanced pelvic organ prolapse. Am J Obstet Gynecol. 2008 May;198(5):573.e1-7.<br>21) Nelson G, Bakkum-Gamez J, Kalogera E, Glaser G, Altman A, Meyer LA, Taylor JS, Iniesta M, Lasala J, Mena G, Scott M, Gillis C, Elias K, Wijk L, Huang J, Nygren J, Ljungqvist O, Ramirez PT, Dowdy SC. Guidelines for perioperative care in gynecologic/oncology: Enhanced Recovery After Surgery (ERAS) Society recommendations-2019 update. Int J Gynecol Cancer. 2019 Mar 15. pii: ijgc-2019-000356.

Annex 1 - ERAS protocol for the perioperative management of patients undergoing hysterectomy for benign or malignant gynecological pathology.

Inclusion criteria

- The ERAS protocol is applied to all patients who are candidates for elective hysterectomy surgery for benign gynecological pathology or for neoplasia of the neck or body of the uterus.

Exclusion criteria

- ASA V.
- Patients undergoing an emergency hysterectomy.
- Patient candidates for hysterectomy surgery for pelvic floor disease.

Pre-operative evaluation and information

- Anesthetic evaluation
Anesthetic visit to be performed at least 2 weeks before surgery to stabilize any clinical conditions: cardiological diseases, anemia, COPD, diabetes, states of nutritional deficiency. Also invite with adequate support to abstain from smoking and alcohol. If the patient has a history of severe respiratory disease (COPD, asthma, sleep apnea syndrome), with an uncompensated clinical picture, it is indicated to request a clinical-instrumental evaluation of the respiratory function, aimed at identifying the subjects who could benefit from pre- and / or postoperative physiotherapy treatment.

- Preoperative counseling
A meeting must be provided between the patient and the multidisciplinary team (surgeon, anesthetist and nurse). The aim is to promote compliance with the protocol by sharing the objectives with the patient and motivating her to adhere to the path outlined. To this end, the involvement of family members who will participate in the preoperative counselling and assist the patient both during hospitalization and once back home is useful. Counseling should take place sufficiently in advance (indicatively at least 2 weeks) with respect to the expected date of admission. The meeting must possibly be conducted in a multidisciplinary context, with the simultaneous participation of all the professionals involved. This makes it possible to share the contents of health education and information that the patient must receive, to avoid repetitions and optimally finalize the counselling. Anesthetist and surgeon inform the patient about the procedures within their competence and obtain informed consent. The nurse has the task of:

- carrying out an assessment of the patient and family members needs (including nutritional risk);
- inform the patient about the organization of the ward, the staff and the necessary items;
- inform the patient on preparation (nutrition, supplement intake, mobilisation program) and on the management of pain and any postoperative nausea / vomiting.

It is advisable that the verbal information is integrated with the delivery of the specially prepared information material (information leaflet in annex 5).

-Assessment of nutritional status and dietary prescriptions

- A preoperative nutritional risk assessment must be performed using the Malnutrition Universal Screening Tool (Annex 6).

- In patients with a MUST score ≥ 1, an evaluation by the Dietetics and Clinical Nutrition Service is indicated for the evaluation of nutritional status and for dietary therapy, including immunonutrition.
- For the diagnosis of malnutrition, use the GLIM criteria identified in the consensus document of ESPEN 2018 (annex 7).
- Preoperative administration of immunonutrition for 5-7 days before surgery is indicated in all malnourished patients. The preoperative immunonutrition treatment consists in the administration of 2 bricks for about 450-500 ml of an oral nutritional supplement enriched with immunonutrients (arginine, w-3 fatty acids and RNA).
- In malnourished patients it is necessary that the nutritional evaluation take place at least 10 days before the operation; in case of severe malnutrition it may be necessary to postpone surgery.
- To assess the compliance of patients with immunonutrition, use the form for monitoring the intake of the preoperative nutritional supplement (annex 8).
- No food restrictions up to 6 hours before the surgery; with the possibility of taking clear liquids up to 2 hours before surgery.
- Carbohydrate load: administration of a maltodextrin-based drink, free of lipids, lactose, fiber and gluten. Dose: 800 cc the evening before the surgery and 400 cc 2-3 hours before the surgery, then fasting. The drink should be taken fresh if possible.
- The intake of the maltodextrin-based drink is contraindicated in patients with slow gastric emptying (obesity, decompensated diabetes, achalasia or history of hiatal hernia) or in patients with difficult management of the airways. In diabetic patients it is not contraindicated, but in the case of an uncompensated clinical picture it is advisable the evaluation by the specialist.
- The administration of the maltodextrin-based drink in the dose of 800 cc the day before the surgery will not be carried out in patients undergoing preoperative immunonutrition, as these patients will continue to take the oral nutritional supplement enriched with immunonutrients.

-Colic preparation:
No colic preparation.

- Antithrombotic prophylaxis according to ERAS 2019 guidelines
Positioning of elastic compression stockings + LMWH at prophylactic dose in patients with the following risk factors who undergo surgery whose expected duration is more than 30min:

- BMI> 35
- Age> 65 years
- Pre-operative therapy with corticosteroids or hormones
- Previous chemotherapy
- Bed rest and poor mobilization
- History of previous DVT / TEP
- Compression of the tumor mass on the vessels

- Antibiotic prophylaxis according to the ERAS 2019 guidelines
The use of first generation cephalosporins is recommended within one hour of the start of surgery with the addition of coverage on anaerobes in case of surgery involving the intestine and in surgery for tumors of the pelvis.
Repetition of the antibiotic dose should be based on the duration of the intervention and on blood loss.

-Prevention of anemia:

Before surgery, attempts should be made to correct the anemia according to the guidelines or current clinical practice of the centre. Intravenous iron preparations are preferred over oral iron to restore hemoglobin concentrations more rapidly in both iron deficiency anemia and chronic disease anemia. Blood transfusion should be avoided if possible.

Anesthetic protocol

- No routine pre-anesthesia, in particular avoiding the administration of long-acting benzodiazepines.
- Prevention of hypothermia and constant monitoring of body temperature, pre-warming recommended.
- Glucose control: Glucose values should be maintained <200 mg / dl in diabetic and non-diabetic patients.

- In laparotomy surgery the protocol requires the placement of an epidural catheter (indicatively T10-T11 or T11-T12) before the induction of general anesthesia. Administer verification test dose and initial bolus of local anesthetic.
- In laparoscopic surgery the placement of the epidural catheter is at the discretion of the anesthetist.
- In laparoscopic surgery and in cases where it is not indicated to use the epidural catheter in laparotomy surgery, the protocol require:
  - Ultrasound-guided bilateral tap block after induction of anesthesia with Ropivacaine 0.25% 100-150 mg depending on the patient's weight.
  - spino-analgesia with Morphine 0.10 -0.15 mg in total 3 ml of 0.9% NaCl before induction.
  - Infiltration of the surgical wound with long-acting local anesthetic.
- Inhaled general anesthesia or TIVA, induction and maintenance with short-acting drugs and curarization. The use of monitors to measure depth of anesthesia and curarization is recommended (deep block in laparoscopic surgery, use of reversal curar antagonists is recommended at the end of anesthesia).
- In vaginal hysterectomy, rachianesthesia with 0.5% hyperbaric bupivacaine or 0.5% levobupivacaine is recommended whenever possible.
- Protective ventilation is recommended (VT 6-8ml / kg with PEEP 6-8cm H_2O)
- Restrictive intraoperative hydration,1-4ml/kg/h of crystalloid solutions. Maintain an intraoperative diuresis of at least 0.5ml/kg/h. Use Goal Directed Fluid Therapy (GDFT) in high-risk patients.
- The use of vasoconstrictors is recommended in hypotensive normovolaemic patients under treatment with epidural analgesia.
- Prevention of hypothermia and constant monitoring of body temperature.
- Selective emesis prophylaxis:
  - Apfel score 1-2, prophylaxis with 2 first-line drugs;
  - Apfel score ≥ 3, prophylaxis with 2-3 antiemetic drugs.

  Please note that patients undergoing hysterectomy start with a base score of 2, therefore prophylaxis with at least 2 antiemetic drugs (Ondansetron 4mg + Dexamethasone 4mg / 8mg) is recommended to be repeated at 12 and 24 hours
- Positioning of the bladder catheter to be removed as early as possible in the postoperative period.
- No nasogastric tube positioning or to be limited to the intraoperative period.

Surgical technique

- The laparoscopic or vaginal approach is to be preferred where there is adequate experience of the operators and obviously it is technically applicable to the patient.
- Abstention from routine use of abdominal drains

Post-operative management

- Removal of the nasogastric tube upon awakening (if positioned).

- Immediate postoperative monitoring:
    - vital signs (PAO, HR, SpO2) and diuresis,
    - pain assessment,
    - body temperature control.

- Infusion therapy:
    - post-operative fluid infusion: 1-2ml /kg/h in the first 24 hours;
    - removal of intravenous infusions within the first postoperative day.

- Early feeding:
    - two hours after waking up from anesthesia reintroduction of the water diet;
    - administration of the meal after 6-8 hours from the end of the surgery, according to the patient's tolerance;
    - use of liquid or creamy caloric-protein oral supplements: suggested until caloric and protein requirements are reached; for malnourished patients the administration of immunonutrition for 5 days post surgery is indicated;
    - It is useful for the patient to keep a diary to record the intake of drinks and food after surgery (Annex 9).

- Early mobilization
    - on the day of surgery: mobilize (sitting) 4 hours after waking up with the aim of remain seated for 2 hours;
    - 1st day: the patient's goal is to stay out of bed at least 8 h and walk;
    - 2nd day: normal activity, not less than what described for the 1st day.

    It is recommended to use suitable areas and armchairs to facilitate the stay out of bed.

- Early removal of the bladder catheter. The bladder catheter must be removed at the beginning of the 1st postoperative day. The catheter should be kept in case of diuresis <500 ml/24 h.

- Postoperative analgesia
    - In patients with a functioning epidural catheter with continuous infusion of ropivacaine 0.125-0.2% +/- addition of short-acting opioid (usually up to the 2nd day) analgesia should be supplemented if necessary with paracetamol 1 g iv (max 4g / die).
    - Removal of the epidural catheter if NRS <5 for at least 4-6 h from the suspension of the epidural administration, followed by oral analgesic control with paracetamol and NSAIDs as needed.
    - If the epidural catheter has not been positioned, NSAIDs + paracetamol and possibly minor opioids are prescribed - IV on day 0 and orally from the first day.

- We do not recommend the use of major opioids which, having dose-dependent side effects, could delay early refeeding and increase the chances of postoperative ileus.

Daily survey of the "fit for discharge" criteria

1. Oral nutrition is tolerated.
2. Resumption of intestinal function (sufficient passage of flatus).
3. Pain control with oral analgesics.
4. Mobility autonomy and personal hygiene care.
5. No clinical / laboratory evidence of postoperative complications
6. Patient's consent at discharge.

Post-discharge checks

- Telephone contact with the patient 2-3 days post-discharge.
- Post-surgical visit in the 30-40 days following the operation.

Annex 2 - Data collection form (Case Report Form - CRF).

PDF file attached

Annex 3 - QoR-15 questionnaire to evaluate the quality of post-operative recovery.

PDF file attached

Annex 4: SSQ-8 questionnaire to assess patient satisfaction after discharge - Italian version obtained through a translation and backward translation process ("forward / backward translation"), to be subjected to validation on a first sample of patients.

ERAS study
Gynecological surgery
Questionnaire to evaluate satisfaction after surgery

Instructions: Here are some questions relating to the degree of satisfaction with the surgery you have recently undergone. Please indicate which of the answers best expresses your experience. All responses are strictly confidential and covered by EU regulation 269/2016.

1. How satisfied are you with how your pain was controlled after the surgery while you were in the hospital?

□ very satisfied □ satisfied □ partially satisfied □ dissatisfied □ very dissatisfied

2. How satisfied are you with how your pain was controlled once you got home after the surgery?

□ very satisfied □ satisfied □ partially satisfied □ not satisfied □ very dissatisfied

3. How satisfied are you with the time you have taken to resume your daily activities, for example doing housework or social activities outside the home?

□ very satisfied □ satisfied □ partially satisfied □ not satisfied □ very dissatisfied

4. If you are working (otherwise skip the question), how satisfied are you with the time it took you to get back to your job?

□ very satisfied □ satisfied □ partially satisfied □ not satisfied □ very dissatisfied

5. How satisfied are you with the time it took you to resume your normal physical activity?

□ very satisfied □ satisfied □ partially satisfied □ not satisfied □ very dissatisfied

6. How satisfied are you with the assistance received?

□ very satisfied □ satisfied □ partially satisfied □ not satisfied □ very dissatisfied

7. How satisfied are you with the information received from the healthcare staff (doctor or nurse) about your intervention?

□ very satisfied □ satisfied □ partially satisfied □ not satisfied □ very dissatisfied

8.Would you recommend other people with the same problem to have surgery in the same hospital?

□ yes □ maybe □ I don't know □ I don't think so □ no

Annex 5 - Information leaflet for the patient.

PDF file attached

Annex 6 - MUST score

Available at: https://www.bapen.org.uk/pdfs/must/must_full.pdf

Annex 7 - ESPEN 2018 consensus document

PDF file attached

Annex 8: Example of form for monitoring the intake of the preoperative nutritional supplement

PDF file attached

Annex 9: Example of diary of the patient for monitoring mobilization and post-operative food and beverage intake.

PDF file attached
