# Supplementary material for: Implementation of the ERAS (Enhanced Recovery After Surgery) protocol for hysterectomy in the Piedmont Region with an audit&feedback approach: Study protocol for a stepped wedge cluster randomized controlled trial. A study of the EASY-NET project
Source: PLoS One. 2022 May 27;17(5):e0268655. doi: 10.1371/journal.pone.0268655 (PMC9140274; doi:10.1371/journal.pone.0268655)
Supplement: S3 File — (PDF) [file pone.0268655.s003.pdf]

**Titolo**	**Studio controllato randomizzato a cluster - stepped wedge - sull' implementazione del protocollo ERAS (Enhanced Recovery After Surgery) nella gestione perioperatoria delle pazienti sottoposte a isterectomia per patologia ginecologica benigna o per neoplasie del collo-corpo dell'utero in Regione Piemonte. Uno studio del progetto EASY-NET.**
**Titolo breve**	**Progetto ERAS Isterectomia**
**Versione**	2.0 (gennaio 2020)
**Promotore**	ASO Santa Croce e Carle di Cuneo<br>SC Ostetricia e Ginecologia
**Funding**	Lo studio è incluso tra le attività del Programma di Rete della Ricerca Sanitaria Finalizzata del Ministero della Salute Anno 2016 (NET-2016-02364191) finanziato dal Ministero della Salute e dalla Regione Piemonte (EASY-NET).

**Principal investigators**	Marco Camanni, MD<br>Ospedale Martini – ASL Città di Torino, Torino<br>Responsabile Area Chirurgia Ginecologica POIS<br><br>Andrea Puppo, MD<br>Ospedale S. Croce e Carle, Cuneo (centro promotore e coordinatore dello studio)<br>Direttore SC Ginecologia e Ostetricia<br><br>Giovannino Ciccone, MD PhD<br>Unità di Epidemiologia Clinica e Valutativa, "Città della Salute e della Scienza" di Torino e CPO Piemonte, Torino<br>PI del WP3 del programma di rete della RSF2016 (EASY-NET)
**Comitato scientifico**	Elena Maria Delpiano, MD<br>SC Ostetricia e Ginecologia<br>Ospedale Martini – ASL Città di Torino, Torino<br><br>Laura Ceretto Giannone, MD<br>Servizio di Anestesia e Rianimazione<br>Ospedale Gradenigo Humanitas, Torino<br><br>Eva Pagano, MSc<br>Unità di Epidemiologia Clinica e Valutativa, "Città della Salute e della Scienza" di Torino e CPO Piemonte, Torino<br>coPI del WP3 del programma di rete della RSF2016 (EASY-NET)<br><br>Elisa Piovano, MD PhD<br>SC Ostetricia e Ginecologia<br>Ospedale Regina Montis Regalis, Mondovì (CN)<br><br>Andrea Scoletta, MD<br>SC Ostetricia e Ginecologia<br>Ospedale Santa Croce, Moncalieri (TO)<br><br>Paolo Zola, MD<br>Città della Salute e della Scienza di Torino, Ospedale S.Anna, Torino<br>Coordinatore gruppo di studio Tumori Ginecologici - Rete Oncologica Piemonte Valle d'Aosta<br><br>Monica Rolfo<br>Responsabile Infermieristico Struttura Humanitas Cellini e Clinca Fornaca Sessant, Torino<br>Referente Infermieristico Nazionale POIS per il quadriennio 2016/2019<br><br>Anna Maria Orlando, MA<br>Direzione Sanità<br>Settore Assistenza Specialistica ed Ospedaliera<br>Regione Piemonte<br><br>Oscar Bertetto; MD, PhD<br>Direttore Dipartimento Interaziendale Interregionale Rete Oncologica del Piemonte e Valle d'Aosta, AOU Città della Salute e della Scienza di Torino.

**Indice**	Background<br>Ipotesi<br>Obiettivo principale<br>Obiettivi secondari<br>Disegno dello studio<br>Criteri di inclusione<br>Criteri di esclusione<br>Metodi di stratificazione e randomizzazione dei centri<br>Gestione peri-operatoria standard<br>Gestione sperimentale (ERAS)<br>Endpoint<br>Metodi e strumenti di raccolta dei dati e di feedback<br>Considerazioni statistiche su numerosità e potenza dello studio<br>Analisi Statistiche<br>Bibliografia	Pag. 4<br>5<br>6<br>6<br>6<br>6<br>7<br>7<br>7<br>7<br>8<br>8<br>9<br>10<br>11
**Allegati**	1) Protocollo ERAS per la gestione perioperatoria delle pazienti sottoposte a isterectomia per patologia ginecologica benigna o maligna<br>2) Scheda raccolta dati (Case Report Form – CRF)<br>3) Questionario QoR-15 per valutare la qualità del recupero post-operatorio<br>4) Questionario SSQ-8 per valutare la soddisfazione delle pazienti dopo la dimissione<br>5) Opuscolo informativo per la paziente<br>6) MUST score<br>7) Documento di consenso dell'ESPEN 2018<br>8) Esempio di scheda per il monitoraggio dell'assunzione del supplemento nutrizionale preoperatorio<br>9) Esempio di diario del paziente per il monitoraggio della mobilizzazione e della assunzione di bevande ed alimenti post-intervento	13<br>18<br>19<br>20<br>21<br>22<br>23<br>24<br>25

**Background**	Il protocollo ERAS (Enhanced Recovery After Surgery, migliore recupero dopo la chirurgia) si riferisce ad un certo numero di interventi perioperatori che mirano ad attenuare lo stress chirurgico, a ottimizzare la gestione del dolore e a mantenere quanto più possibile la normale fisiologia del paziente (1-2).<br>I principali obiettivi di ERAS sono:<br>• ottimizzare la gestione perioperatoria utilizzando procedure basate sull'evidenza scientifica<br>• favorire un migliore recupero dell'autonomia nel post operatorio<br>• favorire una diminuzione dei tempi di ricovero<br>• aumentare il livello di soddisfazione dei pazienti in merito alle cure ricevute<br>• ridurre l'incidenza di complicanze, riammissioni ospedaliere e costi (3-5).<br><br>Dopo la pubblicazione di numerosi studi in ambito di chirurgia generale (6-7), i protocolli ERAS si stanno diffondendo, guadagnando un crescente interesse anche in ambito ginecologico, grazie alla pubblicazione nel 2016 di specifiche linee guida da parte della ERAS Society (8-9).<br><br>In ambito ginecologico la strategia ERAS prevede un accurato colloquio con la paziente nel preoperatorio volto all'interruzione del fumo ed eventualmente delle terapie ormonali, la riduzione del digiuno preoperatorio, l'omissione della preparazione intestinale, la profilassi del tromboembolismo, una corretta profilassi antibiotica, la prevenzione dell'ipotermia intraoperatoria, l'euvolemia perioperatoria, la prevenzione della nausea e del vomito postoperatorio, un uso molto limitato del sondino nasogastrico, la rimozione precoce del catetere urinario, un'analgesia multimodale per ridurre al minimo il consumo di oppiacei, la mobilizzazione precoce postoperatoria e la nutrizione precoce nel post-operatorio, per favorire una rapida ripresa delle funzioni gastro-intestinali (8-9, 21).<br><br>Una recente pubblicazione del Royal College of Obstetricians and Gynecologists (10) esamina i principali elementi della gestione ERAS e conclude che i programmi ERAS offrono cure perioperatorie sicure e di alta qualità, e dovrebbero diventare una pratica standard per tutte le donne sottoposte a chirurgia ginecologica elettiva.<br>Tuttavia, ad oggi, la maggior parte degli studi pubblicati sui benefici di un programma ERAS rispetto alle cure standard in chirurgia ginecologica sono studi di coorte e spesso analizzano l'effetto di singole procedure specifiche della gestione ERAS; solo raramente il "protocollo ERAS" viene testato come un tutt'uno all'interno di un trial randomizzato (11). Le evidenze scientifiche in merito a ERAS in chirurgia ginecologica oncologica sono ancora più rare (12-14).<br>Tutto questo si riflette in una lenta diffusione dei principi ERAS nella pratica clinica.<br>È stata recentemente condotta una survey a livello internazionale (Paesi Scandinavi, Italia, Austria) in merito alla gestione perioperatoria delle pazienti trattate per carcinoma dell'ovaio (dati in via di pubblicazione). La survey ha chiaramente dimostrato che i principi ERAS sono stati recepiti in modo molto disomogeneo a livello europeo, con l'Italia particolarmente in ritardo nell'aggiornamento dei protocolli perioperatori. La survey ha dimostrato inoltre che la centralizzazione della gestione di patologie complesse, come il carcinoma dell'ovaio, favorisce una gestione operatoria aggiornata ed

	evidence based.<br>Una analoga survey è stata condotta anche in Regione Piemonte, per indagare quanto dei principi ERAS fosse già stato recepito nella gestione delle patologie ginecologiche chirurgiche. Nonostante sia emersa una gestione del perioperatorio piuttosto “tradizionale” è stato segnalato da tutti i rispondenti (22/22) un forte interesse nei confronti dell’approccio ERAS e della partecipazione ad iniziative condivise che mirino all’implementazione di tali principi.<br><br>Un programma formale per l’implementazione del protocollo ERAS richiede tre elementi (13-15):<br>i) un protocollo operativo ERAS aggiornato e condiviso<br>ii) un team che lavora per la formazione degli operatori e per aumentare la compliance al protocollo<br>iii) un sistema di audit e feedback (con database dedicato) per verificare la compliance al protocollo e per monitorare gli outcome clinici.<br><br>In Italia la diffusione della strategia ERAS è l’obiettivo della Perioperative Italian Society- P.O.I.S ERAS Italian Chapter (http://perioperativeitaliansociety.org) la cui mission è promuovere la mini-invasività del percorso chirurgico e migliorare la qualità di vita del paziente nel periodo perioperatorio.<br>La Società ha attivato un network di oltre 70 ospedali italiani e ha messo a punto un database per la chirurgia colo-rettale che ha permesso la recente pubblicazione di studi multicentrici (16-18).<br>In ambito ginecologico il protocollo ERAS-POIS, stilato e recentemente rivisto, sarà il riferimento dello studio in oggetto (allegato n.1).<br><br>L’ipotesi di sperimentare l’applicazione del protocollo ERAS per alcuni interventi selezionati (in particolare per la chirurgia colo-rettale e per gli interventi di isterectomia) e di valutarne l’impatto in termini di miglioramento di efficienza e di sicurezza è stata considerata una priorità regionale, tale da includerla nel più generale progetto di rete sulla valutazione di efficacia degli interventi di audit and feedback (Progetto di rete - Ricerca Sanitaria Finalizzata 2016). In questo progetto di rete (NET-2016-02364191), che coinvolge a livello nazionale 7 regioni, con il coordinamento della Regione Lazio, la Regione Piemonte è responsabile del Work Package 3 (WP3) che prevede l’impiego di un disegno controllato e randomizzato a cluster per stimare l’effetto sull’intero sistema ospedaliero di interventi di miglioramento della qualità dell’assistenza in ambito oncologico, con un approccio di audit and feedback intensivo basato su dati raccolti in database dedicati, gestito centralmente, rispetto ad approcci tradizionali di audit basati su iniziative locali o con indicatori centrali che utilizzano solo dati correnti.
**Ipotesi**	Questo studio è stato progettato sulla base di due ipotesi principali:<br>a) il protocollo ERAS ha una elevata probabilità, sulla base delle evidenze disponibili, di introdurre nella pratica clinica procedure con un bilancio favorevole tra benefici e rischi (sia per i pazienti, sia per il personale);<br>b) che la semplice diffusione del protocollo in ospedali selezionati e favorevolmente predisposti al cambiamento avrebbe un impatto limitato sulla qualità complessiva degli interventi su scala regionale, con conseguente accentuazione dell’eterogeneità delle prestazioni tra centri e di riduzione dell’equità tra le pazienti.<br>Tenuto conto delle evidenze disponibili e delle opportunità fornite dal

	contesto regionale, l'Assessorato alla Sanità della Regione Piemonte ha considerato l'implementazione del protocollo ERAS in tutti gli ospedali regionali, nell'ambito di un progetto di ricerca, un obiettivo ad elevata priorità.<br>Sulla base di queste premesse è stato disegnato un progetto di ricerca/intervento che dovrebbe, in un arco di tempo di circa 2 anni, favorire una corretta conoscenza ed adozione sistematica del protocollo ERAS su scala regionale da parte di tutti i centri che eseguono interventi ginecologici.<br>Per poter stimare in modo accurato l'impatto reale del protocollo è stato disegnato uno studio randomizzato a cluster (dove i cluster sono rappresentati dai reparti di ginecologia degli ospedali regionali) con adozione progressiva del protocollo da parte di gruppi di reparti secondo un calendario determinato in modo random (definito "stepped wedge") che al termine dello studio avrà coinvolto tutti i cluster. Al termine dello studio ogni cluster avrà un periodo di attività con procedure abituali ("periodo di controllo") ed uno successivo all'introduzione del protocollo ("periodo sperimentale") analogamente ai disegni con cross-over, ma con una sola transizione (da controllo a sperimentale).<br>Ipotizziamo che l'adozione del protocollo determini una riduzione della durata della degenza, delle complicanze, dei costi sanitari e migliori il recupero funzionale e la soddisfazione delle pazienti.
**Obiettivo principale**	Ridurre la durata della degenza totale nel periodo sperimentale rispetto al periodo di controllo attraverso l'implementazione del protocollo perioperatorio standardizzato ERAS per la gestione delle pazienti candidate a isterectomia (protocollo ERAS-POIS).
**Obiettivi secondari**	Verificare che nel passaggio da periodo di controllo a periodo sperimentale si riduca la frequenza di:<br>• complicanze post chirurgiche<br>• trasferimenti in terapia intensiva<br>• degenze di durata superiore alla soglia<br>• reinterventi<br>• accessi in PS<br>• riammissioni in ospedale<br>Valutare la compliance al protocollo ERAS, complessivamente e per i singoli aspetti/procedure.<br>Comparare tra i due periodi:<br>• l'utilizzo della tecnica laparoscopica.<br>• la qualità del recupero post-operatorio<br>• la soddisfazione percepita delle pazienti<br>• l'impatto economico<br>Nel periodo sperimentale analizzare:<br>• i fattori organizzativi e strutturali dei reparti associati alla compliance al protocollo ERAS<br>• la relazione tra compliance al protocollo ERAS e outcome delle pazienti<br>• la soddisfazione degli operatori.
**Disegno dello studio**	Studio multicentrico controllato randomizzato a cluster, con disegno *stepped wedge*, di confronto tra la gestione perioperatoria standard e quella secondo il protocollo ERAS.
**Criteri di inclusione**	Tutti i reparti di chirurgia ginecologica degli ospedali regionali che eseguono interventi di isterectomia.<br>Tutte le pazienti che durante lo studio sono ricoverate per intervento chirurgico elettivo di isterectomia per patologia ginecologica benigna o per neoplasie del collo o del corpo dell'utero.

**Criteri di esclusione**	Reparti con una casistica inferiore a 20 pazienti anno (meno di 20 interventi di isterectomia/anno).<br>Pazienti candidate a intervento chirurgico di isterectomia per patologia del pavimento pelvico.<br>Pazienti sottoposte a intervento di isterectomia in regime di urgenza.<br>Particolari condizioni di complessità o gravità clinica, da documentare al momento del ricovero, che rappresentino controindicazioni all'applicazione del protocollo ERAS.<br>Pazienti con disagio socio-assistenziale, da documentare al momento del ricovero, che non permettano un adeguato standard di autonomia personale o di assistenza domiciliare post operatoria (condizioni di demenza grave, disfunzione fisica, stato di indigenza) saranno incluse e gestite secondo quanto previsto dallo studio, salvo la decisione della dimissione che dovrà essere valutata in relazione ai problemi specifici (informazioni previste nel database).
**Metodi di stratificazione e randomizzazione dei centri**	In base ad una scelta di tipo programmatorio regionale sono inclusi nello studio tutti i centri accreditati ad effettuare chirurgia ginecologica nella Regione Piemonte che soddisfano i criteri di inclusione specifici dello studio.<br>Prima dell'inizio dello studio tutti i centri saranno contattati dal gruppo di coordinamento per valutare il livello di conoscenza del protocollo ERAS per la chirurgia ginecologica, la predisposizione ad adottarlo (con registrazione delle principali barriere esistenti) o l'eventuale adozione già avvenuta precedentemente.<br>I centri che risultassero avere già adottato pienamente il protocollo prima dell'inizio dello studio saranno esclusi dalla randomizzazione e inclusi in un gruppo osservazionale.<br>Tutti gli altri centri saranno ordinati in base al volume di isterectomie eseguite nel corso del 2017 e suddivisi in 4 strati (con un numero uguale di reparti per strato). Saranno quindi estratti in modo random gruppi di 4 centri (uno per strato) e i gruppi così formati saranno ordinati secondo una sequenza random di periodo di attivazione del protocollo. In questo modo si garantisce una numerosità degli interventi ed una composizione omogenea dei gruppi per ciascun periodo di attivazione. Tutte le procedure di randomizzazione saranno eseguite dopo aver anonimizzato i centri. La data di attivazione del protocollo e dell'evento formativo sarà infine comunicata a ciascun centro con un preavviso di circa 3 mesi (tempo necessario per consentire la formazione del personale e la predisposizione degli aspetti organizzativi locali). Tutte le procedure di randomizzazione saranno eseguite centralmente dalla SSD Epidemiologia Clinica e Valutativa del CPO – Piemonte.
**Gestione peri-operatoria standard**	Prima dell'attivazione del protocollo ciascun centro continuerà a gestire il perioperatorio delle pazienti secondo i protocolli abituali, possibilmente senza introdurre variazioni.
**Gestione sperimentale (ERAS)**	Per ciascun gruppo di circa 4-6 centri nel trimestre precedente la data di attivazione del protocollo verrà effettuata un'approfondita formazione in merito ai principi ERAS e al nuovo protocollo ERAS-POIS (allegato n.1). La formazione si articolerà in un corso della durata di una giornata gestito da esperti formatori POIS.<br>Ciascun centro aderente dovrà identificare, oltre al direttore della struttura e al coordinatore infermieristico, un professionista di riferimento per ciascuna figura professionale coinvolta nel progetto (ginecologo-anestesista-infermiere-dietologo/dietista, medico di direzione sanitaria). Il "TEAM ERAS" regionale parteciperà alla formazione e si renderà successivamente disponibile per approfondimenti e fornire supporto all'implementazione del

	protocollo e alla formazione a livello locale.
**Endpoint**	Primario:<br>• durata media della degenza, calcolata escludendo le durate superiori ad una soglia predefinita di 12 giorni (corrispondente al 98° percentile della distribuzione delle durante di degenza nel 2018)<br>Secondari:<br>• incidenza di complicanze generali e post chirurgiche nel post-operatorio, definite secondo la classificazione Clavien-Dindo<br>• trasferimenti in terapia intensiva nel post-operatorio<br>• percentuale di degenze di durata superiore alla soglia (>12 giorni)<br>• percentuale di reinterventi (entro lo stesso ricovero o comunque nei 30 giorni successivi all'intervento)<br>• percentuale di accessi in PS entro 30 giorni dall'intervento (indipendentemente dal motivo e dall'eventuale ricovero)<br>• percentuale di riammissioni in ospedale entro 30 giorni dall'intervento (indipendentemente dal motivo)<br>• percentuale di interventi eseguiti con tecnica laparoscopica<br>• score di qualità del recupero post-operatorio (misurato con il questionario QoR-15 [19] a circa 24 ore dall'intervento<br>• score di soddisfazione percepita delle pazienti misurato con il questionario SSQ-8 [20] tramite intervista telefonica a due settimane dalla dimissione (solo per un campione di pazienti che accettano di essere intervistate)<br>• valutazione della soddisfazione degli operatori, rilevata in modo qualitativo attraverso focus group, al termine della sperimentazione<br>• costi medi assistenziali, calcolati dal pre-ricovero fino a 30 giorni dopo l'intervento.
**Metodi e strumenti di raccolta dei dati e di feedback**	Prima dell'avvio dello studio tutti i reparti saranno abilitati ad inserire i dati delle pazienti sottoposte ad isterectomia incluse nello studio in un'area dedicata su una piattaforma elettronica (sviluppata e gestita dalla SSD Epidemiologia Clinica e Valutativa - CPO della CSS di Torino). Nel database saranno registrati in modo prospettico e dopo anonimizzazione reversibile i dati relativi alla gestione del peri-operatorio di tutte le pazienti sottoposte ad isterectomia che avranno accettato di rendere disponibili i loro dati ai fini dello studio sottoscrivendo un modulo di consenso, dopo aver ricevuto adeguata informazione scritta ed orale nel periodo del pre-ricovero. La scheda raccolta dati (Case Report Form – CRF) è riportata nell'allegato 2.<br>I componenti del TEAM ERAS di ciascun centro avranno accesso a tale piattaforma attraverso ID e password individuali e potranno visualizzare e modificare soltanto i dati relativi alle proprie pazienti. Il database sarà sviluppato rispettando tutti i requisiti di sicurezza previsti dalla GDPR (legge EU 2016/679) in vigore dal 25/5/2018 e successive modificazioni.<br>Pertanto, dopo l'avvio dello studio (che avverrà contemporaneamente in tutti i centri), ciascun centro registrerà sulla piattaforma web i dati relativi alla gestione standard delle proprie pazienti candidate a isterectomia. Successivamente, in base al calendario di attivazione del protocollo ERAS in ciascun centro, il team abilitato del reparto avrà anche accesso alla sezione di monitoring dell'area nella quale saranno disponibili, con aggiornamento contemporaneo al caricamento dei dati, grafici con i principali endpoint dello studio, informativi sull'andamento generale del protocollo a livello regionale e del centro specifico. Questa forma di feedback, centrata su indicatori di processo (aderenza della pratica clinica alle procedure previste nel protocollo

	ERAS) e di esito (durata della degenza, endpoint secondari) dovrebbe consentire a ciascun reparto l'identificazione puntuale e tempestiva delle criticità per poter indirizzare gli eventuali interventi correttivi.<br>Il recupero post-operatorio sarà misurato a circa 24 ore dall'intervento attraverso il questionario QoR-15 (disponibile e validato in lingua Inglese). La versione in italiano è stata ottenuta attraverso un processo di traduzione e retro traduzione ("forward/backward translation") e successiva validazione su un primo campione di pazienti (allegato n.3). A tal fine verrà somministrata alle pazienti anche una visual analog scale (VAS), con scala compresa tra "recupero scadente" e "recupero eccellente", come misura sintetica della qualità del recupero.<br>La soddisfazione percepita dalle pazienti sarà misurata attraverso il questionario SSQ-8 (allegato n.4), somministrato telefonicamente ad un campione di pazienti o loro caregivers dopo due settimane dalla dimissione, da personale esperto nella somministrazione di questionari a contenuto sanitario. Poichè il questionario, validato e disponibile in lingua inglese, non è disponibile in lingua italiana, la versione italiana è stata ottenuta attraverso un processo di traduzione e retro traduzione ("forward/backward translation") e successiva validazione su un primo campione di pazienti, attraverso la correlazione con complicanze, reinterventi, accessi in PS e riammissioni.<br>La valutazione della soddisfazione degli operatori sarà effettuata attraverso questionari e, in modo qualitativo, attraverso la conduzione di focus group, nella fase conclusiva della sperimentazione, coinvolgendo separatamente i diversi gruppi di professionisti (infermieri, ginecologi, anestesisti). I focus group saranno condotti da personale esperto.<br>I costi assistenziali saranno valutati includendo le seguenti categorie di risorse: visite pre-intervento, giornate di degenza (incluse giornate in terapia intensiva), tipologia di intervento, trattamento delle complicanze, reinterventi, accessi in PS, nuovi ricoveri.<br>Per la valutazione dei fattori organizzativi e strutturali dei reparti associati alla compliance al protocollo ERAS si intende somministrare ai centri partecipanti un breve questionario, in concomitanza dello svolgimento dei corsi di formazione, al fine di raccogliere alcune informazioni di contesto (dimensioni del reparto, organizzazione, conoscenza e predisposizione verso il protocollo ERAS, criticità).
**Considerazioni statistiche su numerosità e potenza dello studio**	Tenuto conto che nel 2018 i 22 centri operanti in regione (che non hanno ancora adottato ERAS) hanno eseguito in totale circa 1900 isterectomie programmate, con una media di 22 interventi al trimestre per ciascun centro, ogni gruppo di 4-5 centri dovrebbe eseguire circa 90 interventi al trimestre. Con un calendario di attivazione che prevede un gruppo di 4-5 centri ogni trimestre, sono necessari, dopo un trimestre iniziale, 4 trimestri (15 mesi in totale) per completare l'implementazione delle LG ERAS su tutti i centri della regione.<br>Lo schema della tabella seguente riporta una possibile sequenza di attivazione dei cluster nel corso dello studio con la numerosità dei reparti e delle pazienti per il periodo di controllo e sperimentale. Con questa ipotesi di numerosità attesa (n=2400, di cui 1200 interventi nel periodo di controllo e 1200 nel periodo sperimentale) e di disegno è stata calcolata la potenza statistica dello studio sia per l'endpoint principale (durata della degenza), sia per gli endpoint di sicurezza (frequenza di complicanze, reinterventi).<br><br>Per il calcolo della potenza statistica dello studio è stato applicato il metodo di Hemming e Girling utilizzando il software STATA (v. 13).

	La potenza è stata calcolata ipotizzando che l'applicazione del protocollo comporti una riduzione della durata media della degenza di almeno un giorno (corrispondente ad un effect size di circa 0.5) per essere definito efficace.<br>I parametri utilizzati per il calcolo sono:<br>Degenza media (standard): 4.2 giorni (DS: 2.0)<br>Degenza media (sperimentale): <= 3.2 giorni (DS 2.0)<br>Errore alfa (due code): 0.05<br>Coefficiente di correlazione entro cluster (ICC): 0.10<br>Numerosità media dei cluster: 22<br>Numero di cluster randomizzati per step: 5<br>Numero di step (escluso il baseline): 4<br><br>Numerosità totale dello studio: 2400<br>Potenza statistica: 0.99<br><br>È stata anche calcolata la potenza statistica dello studio per evidenziare come statisticamente significative differenze assolute di almeno 10% degli endpoint secondari misurabili come percentuali (es. aderenza agli items del protocollo ERAS, complicanze, reinterventi, ecc…). Assumendo un valore di riferimento pari a 0.5 (valore più sfavorevole da un punto di vista statistico), e mantenendo tutti i parametri precedenti, lo studio ha una potenza di 0.82.

mesi	0	3	6	9	12	Totale
Step 4	120	120	120	120	120	
Step 3	120	120	120	120	120	
Step 2	120	120	120	120	120	
Step 1	120	120	120	120	120	
Numerosità per periodo:						
Pazienti (di controllo)	480	360	240	120	0	1200
Pazienti (sperimentali)	0	120	240	360	480	1200

**Analisi Statistiche**	Le degenze medie (calcolate escludendo le durate maggiori della soglia) saranno confrontate tra i periodi con e senza implementazione del protocollo ERAS usando modelli di regressione lineare ad effetti casuali. Nel modello, la degenza verrà trattata come variabile dipendente mentre tra le indipendenti una variabile dicotomica indicherà per ciascun centro l'implementazione del protocollo nel periodo di riferimento della degenza. L'effetto dell'implementazione del protocollo ERAS sulla degenza media verrà aggiustato includendo nel modello le variabili tempo (identificato dagli step previsti) e la tecnica chirurgica (LPT, LPS). Il centro verrà incluso nel modello come effetto casuale. Per gli endpoint dicotomici misurati come proporzioni (es. durate di degenza superiori alla soglia, complicanze, riammissioni in ospedale) si stimerà l'effetto dell'implementazione del protocollo ERAS con modelli di regressione logistica ad effetti casuali (centro), utilizzando un indicatore dell'evento (0, 1) come variabile dipendente e includendo nel modello lo stesso set di covariate utilizzato per l'analisi della durata di degenza.<br>Le analisi principali saranno anche stratificate per caratteristiche dei centri (classificati per volume di attività, per grado di aderenza al protocollo ERAS al baseline e per altre caratteristiche strutturali) e delle pazienti (classi di età, diagnosi, tecnica chirurgica utilizzata). Dall'analisi principale saranno esclusi a posteriori i centri con elevata aderenza al protocollo ERAS al baseline.

	Per ridurre il rischio di bias dovuto ad una inclusione selezionata di pazienti nello studio da parte dei centri (selection bias, valutato in base alla percentuale di casi inseriti sul totale delle schede di dimissione dello stesso periodo di arruolamento), si stratificheranno le analisi per completezza dell'arruolamento (con possibilità di esclusione dei centri con maggiore incompletezza).<br>Per tenere conto del periodo di adattamento in ciascun centro è prevista un'analisi di sensibilità che escluderà dal confronto il primo mese di ogni periodo di attivazione del protocollo ERAS.<br>L'impatto del protocollo ERAS sarà anche analizzato in funzione del tempo trascorso dalla sua introduzione per valutare la curva di raggiungimento di un livello accettabile ed ottimale di applicazione.<br>Come analisi secondaria si valuterà l'endpoint principale dello studio sull'intera casistica regionale rilevabile attraverso le schede di dimissione ospedaliera della regione Piemonte, selezionando i pazienti con i criteri di inclusione dello studio.<br>Inoltre, si prevede di valutare l'andamento nel tempo della durata media della degenza rilevabile attraverso le schede di dimissione ospedaliera nei 5 anni precedenti l'attivazione del protocollo ERAS e nell'anno successivo, attraverso un disegno a serie temporali interrotte.
**Bibliografia**	1) Kehlet H and Wilmore DW: Evidence-based surgical care and the evolution of fast-track surgery. Ann Surg 248(2): 189-198, 2008<br>2) Ansari D, Gianotti L, Schroder J and Andersson R: Fast-track surgery: procedure-specific aspects and future direction. Langenbecks Arch Surg 398(1): 29-37, 2013.<br>3) O. Ljungqvist, M. Scott, K.C. Fearon, Enhanced recovery after surgery: a review, JAMA Surg. 152 (3) (2017 Mar 1) 292–298<br>4) Visioni, R. Shah, E. Gabriel, K. Attwood, M. Kukar, S. Nurkin, Enhanced recovery after surgery for noncolorectal surgery?: A systematic review and meta-analysis of major abdominal surgery, Ann. Surg. (2017) (Epub ahead of print).<br>5) O. Ljungqvist, N.X. Thanh, G. Nelson, ERAS - value based surgery, J. Surg. Oncol. (2017 Sep 5) (Epub ahead of print).<br>6) Spanjersberg WR, Reurings J, Keus F and van Laarhoven CJ: Fast track surgery versus conventional recovery strategies for colorectal surgery. Cochrane Database Syst Rev 2, 2011.<br>7) Zhuang CL, Ye XZ, Zhang XD, Chen BC and Yu Z: Enhanced recovery after surgery programs versus traditional care for colorectal surgery: a meta-analysis of randomized controlled trials. Dis Colon Rectum 56(5): 667-678, 2013.<br>8) Nelson G, Altman AD, Nick A, Meyer LA, Ramirez PT, Achtari C, Antrobus J, Huang J, Scott M, Wijk L, Acheson N, Ljungqvist O, Dowdy SC. Guidelines for pre- and intra-operative care in gynecologic/oncology surgery: Enhanced Recovery After Surgery (ERAS) Society recommendations--Part I. Gynecol Oncol. 2016 Feb;140(2):313-22.<br>9) Nelson G, Altman AD, Nick A, Meyer LA, Ramirez PT, Achtari C, Antrobus J, Huang J, Scott M, Wijk L, Acheson N, Ljungqvist O, Dowdy SC. Guidelines for postoperative care in gynecologic/oncology surgery: Enhanced Recovery After Surgery (ERAS) Society recommendations--Part II. Gynecol Oncol. 2016 Feb;140(2):323-32.<br>10) Scientific Impact Paper N°36: Enhanced Recovery in Gynecology, Royal College of Obstetricians and Gynecologists, February 2013 (http://www.rcog.org.uk/files/

rcog-corp/8.2.13SIP36.pdf).

11) Nelson G, Ramirez PT, Ljungqvist O, Dowdy SC. Enhanced Recovery Program and Length of Stay After Laparotomy on a Gynecologic Oncology Service: A Randomize Controlled Trial. Obstet Gynecol. 2017 Jun;129(6):1139.
12) Lindemann K, Kok PS, Stockler M, Jaaback K, Brand A. Enhanced Recovery AftermSurgery for Advanced Ovarian Cancer: A Systematic Review of Interventions
Trialed. Int J Gynecol Cancer. 2017 Jul;27(6):1274-1282.
13) E.Kalogera, J.N. Bakkum-Gamez, C.J. Jankowski, E. Trabuco, J.K. Lovely, S. Dhanorker, P.L. Grubbs, A.L. Weaver, L.R. Haas, B.J. Borah, A.A. Bursiek, M.T. Walsh, W.A. Cliby, S.C. Dowdy, Enhanced recovery in gynecologic surgery, Obstet. Gynecol. 122 (2013) 319–328.
14) E. Miralpeix, A.M. Nick, L.A. Meyer, J. Cata, J. Lasala, G.E. Mena, V. Gottumukkala, M. Iniesta-Donate, G. Salvo, P.T. Ramirez, A call for new standard of care in perioperative gynecologic oncology practice: Impact of enhanced recovery after surgery (ERAS) programs, Gynecol. Oncol. 141 (2) (2016) 371–378.
15) G. Nelson, A.D. Altman. Enhanced recovery after surgery interactive audit system for gynecologic /oncology surgery – importance of measuring protocol element compliance (you don't know what you don't measure!), Gynecol. Oncol. 145 (Suppl. 1) (2017) 191.
16) Braga M, Beretta L, Pecorelli N, Maspero M, Casiraghi U, Borghi F, Pellegrino L, Bona S, Monzani R, Ferrari G, Radrizzani D, Iuliani R, Bima C, Scatizzi M, Missana G, Guicciardi MA, Muratore A, Crespi M, Bouzari H, Ceretti AP, Ficari F; PeriOperative Italian Society Group. Enhanced recovery pathway in elderly patients undergoing colorectal surgery: is there an effect of increasing ages? Results from the perioperative Italian Society Registry. Updates Surg. 2017 Jun
17) Braga M, Borghi F, Scatizzi M, Missana G, Guicciardi MA, Bona S, Ficari F, Maspero M, Pecorelli N; PeriOperative Italian Society. Impact of laparoscopy on adherence to an enhanced recovery pathway and readiness for discharge in elective colorectal surgery: Results from the PeriOperative Italian Society registry. Surg Endosc. 2017 Nov;31(11):4393-4399.
18) Braga M, Pecorelli N, Scatizzi M, Borghi F, Missana G, Radrizzani D; PeriOperative Italian Society. Enhanced Recovery Program in High-Risk Patients Undergoing Colorectal Surgery: Results from the PeriOperative Italian Society Registry. World J Surg. 2017 Mar;41(3):860-867.
19) Stark PA, Myles PS, Burke JA. Development and psychometric evaluation of a postoperative quality of recovery score: the QoR-15. Anesthesiology. 2013 Jun;118(6):1332-40.
20) Murphy M, Sternschuss G, Haff R, van Raalte H, Saltz S, Lucente V. Quality of life and surgical satisfaction after vaginal reconstructive vs obliterative surgery for the treatment of advanced pelvic organ prolapse. Am J Obstet Gynecol. 2008 May;198(5):573.e1-7.
21) Nelson G, Bakkum-Gamez J, Kalogera E, Glaser G, Altman A, Meyer LA, Taylor JS, Iniesta M, Lasala J, Mena G, Scott M, Gillis C, Elias K, Wijk L, Huang J, Nygren J, Ljungqvist O, Ramirez PT, Dowdy SC. Guidelines for perioperative care in gynecologic/oncology: Enhanced Recovery After Surgery (ERAS) Society recommendations-2019 update. Int J Gynecol Cancer. 2019 Mar 15. pii: ijgc-2019-000356.

Allegato 1 - Protocollo ERAS per la gestione perioperatoria delle pazienti sottoposte a isterectomia per patologia ginecologica benigna o maligna.

Criteri di inclusione

- Il protocollo ERAS trova applicazione per tutte le pazienti candidate a chirurgia elettiva di isterctomia per patologia ginecologica benigna o per neoplasia del collo o del corpo dell'utero.

Criteri di esclusione

- ASA V.
- Pazienti sottoposte a intervento di isterectomia in regime di urgenza.
- Pazienti candidate a intervento chirurgico di isterectomia per patologia del pavimento pelvico.

Valutazione e informazione pre-operatoria

- Valutazione anestesiologica
Visita anestesiologica da eseguire almeno 2 settimane prima dell' intervento chirurgico per stabilizzazione delle eventuali condizioni cliniche: malattie cardiologiche, anemia, BPCO, diabete, stati di carenze nutrizionale. Invitare inoltre con adeguato supporto all'astensione dal fumo e alcol. Qualora la paziente presenti un'anamnesi positiva per patologia respiratoria severa (BPCO, asma, sindrome delle apnee notturne), con un quadro clinico non compensato, è indicato richiedere una valutazione clinico-strumentale della funzionalità respiratoria, volta all'identificazione dei soggetti che potrebbero beneficiare di un trattamento fisioterapico pre- e/o postoperatorio.

- Counseling preoperatorio
Dovrà prevedere un momento di incontro tra la paziente e il team multidisciplinare (chirurgo, anestesista e infermiere). Lo scopo è quello di favorire la compliance al protocollo condividendo con la paziente gli obiettivi e motivandola ad aderire al percorso delineato. A tal fine risulta utile il coinvolgimento dei familiari che parteciperanno al colloquio preoperatorio e assisteranno la paziente sia durante la degenza che una volta rientrata al domicilio. E' opportuno che il counseling avvenga con sufficiente anticipo (indicativamente almeno 2 settimane) rispetto alla data prevista del ricovero. L'incontro deve essere condotto possibilmente in ambito multidisciplinare, con la partecipazione contemporanea di tutti i professionisti coinvolti. Ciò consente di condividere i contenuti di educazione sanitaria ed informazione che la paziente deve ricevere, per evitare ripetizioni e finalizzare in maniera ottimale il colloquio. Anestesista e chirurgo informano la paziente sulle procedure di relativa competenza e ottengono il consenso informato. L'infermiere ha il compito di:

- effettuare la valutazione dei bisogni della paziente (incluso il rischio nutrizionale) e dei familiari;
- informare la paziente sull'organizzazione del reparto, sul personale operante e sui presidi necessari;
- informare la paziente sulla preparazione (alimentazione, assunzione degli integratori, programma motorio) e sulla gestione del dolore e di eventuale nausea/vomito postoperatori.

E' consigliabile che l'informazione verbale sia integrata con la consegna del materiale informativo appositamente predisposto (opuscolo informativo in allegato n.5).

-Valutazione dello stato nutrizionale e prescrizioni dietetiche

- Deve essere eseguita una valutazione preoperatoria del rischio nutrizionale utilizzando il Malnutrition Universal Screening Tool (allegato n.6).
- Nelle pazienti con score MUST ≥ 1 è indicata una valutazione del Servizio di Dietetica e Nutrizione Clinica per la valutazione dello stato nutrizionale e per la terapia dietetica, compresa l'immunonutrizione.
- Per la diagnosi di malnutrizione utilizzare i criteri GLIM individuati nel documento di consenso dell'ESPEN 2018 (allegato n.7).
- In tutte le pazienti malnutrite è indicata la somministrazione preoperatoria di immunonutrizione per 5-7 giorni prima dell'intervento. Il trattamento immunonutrizionale preoperatorio consiste nella somministrazione di n°2 briks per circa 450-500 ml di supplemento nutrizionale orale arricchito di immunonutrienti (arginina, acidi grassi w-3 e RNA).
- Nelle pazienti malnutrite è necessario che la valutazione nutrizionale avvenga almeno 10 giorni prima dell'intervento; in caso di malnutrizione severa può essere necessario posticipare l'intervento chirurgico.
- Per valutare la compliance delle pazienti all'immunonutrizione utilizzare la scheda per il monitoraggio dell'assunzione del supplemento nutrizionale preoperatorio (allegato n.8).
- Nessuna restrizione alimentare fino a 6 ore prima dell'intervento; con possibilità di assumere liquidi chiari fino a 2 ore prima dell'intervento.
- Carico glucidico: somministrazione di bevanda a base di maltodestrine e priva di lipidi, lattosio, fibre e glutine. Dose: 800 cc la sera precedente l'intervento e 400 cc 2-3 ore prima dell'intervento, poi digiuno. La bevanda va assunta possibilmente fresca.
- L'assunzione della bevanda a base di maltodestrine è controindicata Nelle pazienti con svuotamento gastrico lento (obesità, diabete scompensato, acalasia o storia di ernia jatale) o pazienti nelle quali sia prevista una difficile gestione delle vie aeree. Nelle pazienti diabetiche non è controindicata, ma in caso di quadro clinico non compensato è opportuna la valutazione da parte dello specialista.
- La somministrazione della bevanda a base di maltodestrine nella dose di 800 cc il giorno precedente l'intervento non sarà effettuata nelle pazienti sottoposte ad immunonutrizione preoperatoria, in quanto queste pazienti continueranno l'assunzione del supplemento nutrizionale orale arricchito di immunonutrienti.

-Preparazione colica:

Nessuna preparazione colica.

-Profilassi antitrombotica secondo le linee guida ERAS 2019

Posizionamento calze elasto-compressive + EBPM a dose profilattica nelle pazienti con seguenti fattori di rischio che vengano sottoposte a intervento la cui durata prevista sia di più di 30min:

- BMI >35
- Età >65 anni
- Terapia pre-operatoria con corticosteroidi o ormonale
- Precedente chemioterapia
- Allettamento e scarsa mobilizzazione
- Storia di pregressa TVP/TEP
- Compressione della massa tumorale sui vasi

-Profilassi antibiotica secondo le linee guida ERAS 2019

Si raccomanda l'utilizzo di Cefalosporine di prima generazione entro un'ora dall'inizio dell'intervento chirurgico con l'aggiunta di copertura sugli anaerobi in caso di chirurgia che interessi l'intestino e negli interventi chirurgici per tumori della pelvi.

La ripetizione della dose antibiotica deve basarsi sui tempi di durata dell'intervento e sull'eventuale perdita ematica.

-Prevenzione dell'anemia:
Prima dell'intervento chirurgico è opportuno tentare di correggere l'anemia secondo le linee guida o la pratica clinica corrente dell'azienda. I preparati di ferro per via endovenosa sono da preferirsi al ferro orale per ripristinare più rapidamente le concentrazioni di emoglobina, sia nell'anemia da carenza di ferro che nell'anemia da malattia cronica. La trasfusione di sangue dovrebbe essere evitata, se possibile.

Protocollo anestesiologico

- Nessuna preanestesia di principio, evitando in particolare la somministrazione di benzodiazepine long acting.
- Prevenzione dell'ipotermia e costante monitoraggio della temperatura corporea, raccomandato il pre-riscaldamento.
- Controllo della glicemia: i valori della glicemia devono essere mantenuti < a 200 mg/dl nelle pazienti diabetiche e non.

- Nella chirurgia laparotomica il protocollo prevede il posizionamento di catetere epidurale (indicativamente T10-T11 o T11-T12) prima dell'induzione dell'anestesia generale. Somministrare dose test di verifica e bolo iniziale di anestetico locale.
- In chirurgia laparoscopica il posizionamento del catetere epidurale è a discrezione dell'anestesista.
- Nella chirurgia laparoscopica e nei casi in cui non sia indicato utilizzare il catetere epidurale in chirurgia laparotomica, il protocollo prevede:
  - Tap block bilaterale ecoguidato dopo l'induzione dell'anestesia con Ropivacaina 0.25% 100-150 mg a seconda del peso della paziente.
  - spino-analgesia con Morfina 0.10 -0.15 mg in 3 ml totali di NaCl 0.9 % prima dell'induzione
  - Infiltrazione della ferita chirurgica con anestetico locale a lunga durata d'azione.
- Anestesia generale inalatoria o TIVA, induzione e mantenimento con farmaci short-acting e curarizzazione. Si raccomanda l'utilizzo di monitoraggi per misurare la profondità dell'anestesia e la curarizzazione (blocco profondo nella chirurgia laparoscopica, si raccomanda l'utilizzo di antagonisti reversal dei curari al termine dell'anestesia).
- Nell'isterectomia vaginale si raccomanda quando possibile rachianestesia con Bupivacaina iperbarica 0.5% o Levobupivacaina 0.5%.
- Si raccomanda ventilazione protettiva (VT 6-8ml/kg con PEEP 6-8 cm H2O)
- Idratazione intraoperatoria restrittiva, 1-4 ml/kg/h di soluzioni cristalloidi. Mantenere una diuresi intraoperatoria di almeno 0.5ml/kg/h. Utilizzare la Goal Directed Fluid Therapy (GDFT) nei pazienti ad alto rischio.
- Si raccomanda l'utilizzo di vasocostrittori nei pazienti normovolemici ipotesi in trattamento con analgesia peridurale.
- Prevenzione dell'ipotermia e costante monitoraggio della temperatura corporea.
- Profilassi emesi selettiva:
  - Apfel score 1-2, profilassi con 2 farmaci di prima linea;
  - Apfel score ≥ 3, profilassi con 2-3 farmaci antiemetici.

  Si ricorda che le pazienti sottoposte ad interventi di isterectomia partono con uno score di base di 2, pertanto si consiglia profilassi con almeno 2 farmaci antiemetici (Ondansetron 4mg + Desametasone 4mg/8mg) da ripetersi a 12 e 24 ore

- Posizionamento catetere vescicale da rimuovere il più precocemente possibile nel post-operatorio.
- Non necessario posizionamento sondino naso-gastrico o da limitare al periodo intraoperatorio.

Tecnica chirurgica

- L'approccio laparoscopico o vaginale è da preferire ove vi sia adeguata esperienza da parte degli operatori e ovviamente sia tecnicamente applicabile alla paziente.
- Astensione dall'uso routinario di drenaggi addominali

Gestione post-operatoria

- Rimozione sondino naso-gastrico al risveglio (qualora posizionato).

- Monitoraggio nell'immediato postoperatorio:
    - parametri vitali (PAO, FC, SpO2) e diuresi
    - valutazione del dolore
    - controllo della temperatura corporea.

- Terapia infusionale:
    - infusione di liquidi post operatoria: 1-2ml/kg/h nelle prime 24 ore;
    - rimozione delle infusioni endovenose entro la prima giornata post-operatoria.

- Alimentazione precoce:
    - due ore dopo il risveglio dall'anestesia reintroduzione della dieta idrica;
    - somministrazione del pasto dopo 6-8 ore dalla fine dell'intervento in base alla tolleranza della paziente;
    - uso di integratori orali calorico-proteici liquidi o cremosi: suggerito fino al raggiungimento dei fabbisogni calorici e proteici; per le pazienti malnutrite è indicata la somministrazione di immunonutrizione per 5 giorni post intervento;
    - è utile che la paziente tenga un diario in cui registrare l'assunzione di bevande e alimenti post-intervento (allegato n.9).

- Mobilizzazione precoce
    - il giorno dell'intervento: dopo 4 ore dal risveglio mobilizzare (seduta) con obiettivo di stare per 2 ore seduta;
    - 1^ giornata: obiettivo per la paziente è restare fuori dal letto almeno 8 h e camminare;
    - 2^ giornata: attività normale, non inferiore a quanto descritto per la 1^ giornata.

  Si raccomanda di utilizzare locali e poltrone adeguati per favorire la permanenza fuori dal letto.

- Rimozione precoce catetere vescicale. Il catetere vescicale va rimosso all'inizio della 1^ giornata post operatoria. Il catetere va mantenuto in caso di diuresi < 500 ml/24 h.

- Analgesia postoperatoria
    - Nelle pazienti con catetere epidurale funzionante con infusione continua di Ropivacaina 0.125-0.2 % +/- aggiunta di oppioide short acting (di regola fino

alla 2^ giornata) l'analgesia va integrata se necessario con paracetamolo 1 g ev (max 4g/die).

- Rimozione del catetere peridurale se NRS<5 per almeno 4-6 h dalla sospensione della somministrazione peridurale, prosegue controllo antalgico per via orale con Paracetamolo e FANS al bisogno.
- Se il catetere epidurale non è stato posizionato si prescrivono FANS + paracetamolo ed eventualmente oppioidi minori - ev in giornata 0 e per os dalla prima giornata.
- Si sconsiglia l'uso di oppioidi maggiori che presentando effetti collaterali dose dipendente potrebbero ritardare una precoce rialimentazione ed aumentare le probabilità di ileo post-operatorio.

Rilevazione giornaliera dei criteri "fit for discharge"

1. Alimentazione orale tollerata.
2. Ripresa funzione intestinale (sufficiente canalizzazione gas).
3. Controllo del dolore con analgesici per os.
4. Autonomia motoria e nelle cure igieniche personali.
5. Non evidenza clinica / laboratoristica di complicanze postoperatorie
6. Consenso della paziente alla dimissione.

Controlli post-dimissione

- Contatto telefonico con la paziente 2-3 giorni post-dimissione.
- Visita postchirurgica nei 30-40 gg successivi all'intervento.

Allegato 2 - Scheda raccolta dati (Case Report Form – CRF).

File pdf allegato

Allegato 3 - Questionario QoR-15 per valutare la qualità del recupero post-operatorio.

File pdf Allegato

Allegato 4: Questionario SSQ-8 per valutare la soddisfazione delle pazienti dopo la dimissione - Versione in italiano ottenuta attraverso un processo di traduzione e retro traduzione ("forward/backward translation"), da sottoporre a validazione su un primo campione di pazienti.

Studio ERAS
Chirurgia ginecologica
Questionario per valutare la soddisfazione dopo un intervento chirurgico

Istruzioni: di seguito sono elencate alcune domande relative al grado di soddisfazione per l'intervento chirurgico al quale si è sottoposta recentemente. Per piacere, indichi quale delle risposte esprime meglio la sua esperienza. Tutte le risposte sono strettamente confidenziali e coperte dal regolamento EU 269/2016.

1. Quanto è soddisfatta di come è stato controllato il suo dolore dopo l'intervento mentre era in ospedale?
   ☐ molto soddisfatta ☐ soddisfatta ☐ parzialmente soddisfatta ☐ insoddisfatta ☐ molto insoddisfatta

2. Quanto è soddisfatta di come è stato controllato il suo dolore una volta tornata a casa dopo l'intervento?
   ☐ molto soddisfatta ☐ soddisfatta ☐ parzialmente soddisfatta ☐ non soddisfatta ☐ molto insoddisfatta

3. Quanto è soddisfatta del tempo che ha impiegato per riprendere le sue attività quotidiane, per esempio svolgere lavori domestici o attività sociali fuori casa?
   ☐ molto soddisfatta ☐ soddisfatta ☐ parzialmente soddisfatta ☐ non soddisfatta ☐ molto insoddisfatta

4. Se lavora (altrimenti saltare la domanda), quanto è soddisfatta del tempo che ha impiegato per tornare al suo lavoro?
   ☐ molto soddisfatta ☐ soddisfatta ☐ parzialmente soddisfatta ☐ non soddisfatta ☐ molto insoddisfatta

5. Quanto è soddisfatta del tempo che ha impiegato per riprendere la sua normale attività fisica?
   ☐ molto soddisfatta ☐ soddisfatta ☐ parzialmente soddisfatta ☐ non soddisfatta ☐ molto insoddisfatta

6. Quanto è soddisfatta dell'assistenza ricevuta?
   ☐ molto soddisfatta ☐ soddisfatta ☐ parzialmente soddisfatta ☐ non soddisfatta ☐ molto insoddisfatta

7. Quanto è soddisfatta delle informazioni ricevute dal personale sanitario (medico o infermieristico) sul suo intervento?
   ☐ molto soddisfatta ☐ soddisfatta ☐ parzialmente soddisfatta ☐ non soddisfatta ☐ molto insoddisfatta

8. Consiglierebbe ad altre persone con lo stesso problema di farsi operare nello stesso ospedale?
   ☐ sì ☐ forse ☐ non so ☐ non credo ☐ no

Allegato 5 - Opuscolo informativo per la paziente.

File allegato .pdf

Allegato 6 - MUST score

available at: https://www.bapen.org.uk/images/pdfs/must/italian/must-toolkit.pdf

Allegato 7 - Documento di consenso dell'ESPEN 2018

File allegato .pdf

Allegato 8: Esempio di scheda per il monitoraggio dell'assunzione del supplemento nutrizionale preoperatorio

File allegato .pdf

Allegato 9: Esempio di diario del paziente per il monitoraggio della mobilizzazione e della assunzione di bevande ed alimenti post-intervento

File allegato .pdf
