# Supplementary material for: Implementation of the ERAS (Enhanced Recovery After Surgery) protocol for hysterectomy in the Piedmont Region with an audit&feedback approach: Study protocol for a stepped wedge cluster randomized controlled trial. A study of the EASY-NET project
Source: PLoS One. 2022 May 27;17(5):e0268655. doi: 10.1371/journal.pone.0268655 (PMC9140274; doi:10.1371/journal.pone.0268655)
Supplement: Attachment — Submitted filename: Response to Reviewers 141121.docx [file pone.0268655.s004.docx]

**Response to the Academic Editor and Reviewers**

Journal requirements:
When submitting your revision, we need you to address these additional requirements.

1. Please ensure that your manuscript meets PLOS ONE's style requirements, including those for file naming. The PLOS ONE style templates can be found at

https://journals.plos.org/plosone/s/file?id=wjVg/PLOSOne_formatting_sample_main_body.pdf and https://journals.plos.org/plosone/s/file?id=ba62/PLOSOne_formatting_sample_title_authors_affiliations.pdf.

***Answer: the manuscript was updated according to PLOS ONE's style requirements.***

2. We note that the grant information you provided in the ‘Funding Information’ and ‘Financial Disclosure’ sections do not match. When you resubmit, please ensure that you provide the correct grant numbers for the awards you received for your study in the ‘Funding Information’ section.

***Answer: the ‘Funding Information’ section should be the following: “This work was supported by the Italian Ministry of Health and the Piedmont Region as part of the Easy-Net Project, grant number NET-2016-02364191”***

3. Thank you for stating the following in the Acknowledgments/ Funding Section of your manuscript:

“This work was supported by the Italian Ministry of Health and the Piedmont Region as part of the Easy-Net Project, grant number NET-2016-02364191.”

We note that you have provided funding information that is not currently declared in your Funding Statement. However, funding information should not appear in the Acknowledgments section or other areas of your manuscript. We will only publish funding information present in the Funding Statement section of the online submission form.

Please remove any funding-related text from the manuscript and let us know how you would like to update your Funding Statement. Currently, your Funding Statement reads as follows:

“The authors have declared that no competing interests exist.”

Please include your amended statements within your cover letter; we will change the online submission form on your behalf.

***Answer: The Funding statement “This work was supported by the Italian Ministry of Health and the Piedmont Region as part of the Easy-Net Project, grant number NET-2016-02364191” was completely removed from the manuscript.***

***The Funding Statement of the online submission form should be updated as follows: “This work was supported by the Italian Ministry of Health and the Piedmont Region as part of the Easy-Net Project, grant number NET-2016-02364191”. Thank you.***

3. One of the noted authors is a group or consortium ERAS-Gyneco Piemonte study group. In addition to naming the author group, please list the individual authors and affiliations within this group in the acknowledgments section of your manuscript. Please also indicate clearly a lead author for this group along with a contact email address.

***Answer: the individual authors and affiliations within the ERAS-Gyneco Piemonte study group are now listed in the acknowledgments section of the manuscript and the lead author (Eva Pagano) and her email are highlighted.***

4. Your ethics statement should only appear in the Methods section of your manuscript. If your ethics statement is written in any section besides the Methods, please delete it from any other section.

***Answer: the “Ethics and dissemination” section is now part of the Methods***

Please review your reference list to ensure that it is complete and correct. If you have cited papers that have been retracted, please include the rationale for doing so in the manuscript text, or remove these references and replace them with relevant current references. Any changes to the reference list should be mentioned in the rebuttal letter that accompanies your revised manuscript. If you need to cite a retracted article, indicate the article’s retracted status in the References list and also include a citation and full reference for the retraction notice.

***Answer: the reference list was checked. No changes to the reference list were done.***

Additional Editor Comments (if provided):
The manuscript can be considered for publication after having competed revision as per reviewers requests.

6. Review Comments to the Author

Please use the space provided to explain your answers to the questions above and, if applicable, provide comments about issues authors must address before this protocol can be accepted for publication. You may also include additional comments for the author, including concerns about research or publication ethics.

You may also provide optional suggestions and comments to authors that they might find helpful in planning their study. (Please upload your review as an attachment if it exceeds 20,000 characters)

Reviewer #1: I have no comments to post. It looks well written and conceived. The analysis looks rigorous and satisfactory.

***Answer: thanks to the Reviewer 1 for the complimentary comments.***

Reviewer #2: The manuscript is too long. We suggest to shorten it. Also revise English form
We suggest the authors to introduce a synoptical scheme of the protocol reported in the supplemental material to improve its understanding.

***Answer: we shortened the manuscript moving paragraphs from the “Statistical plan” section of the Manuscript to the “Statistical plan” section of the Supplemental materials. Moreover paragraphs from the “Data collection” section of the Manuscript were moved to the “Data collection” section of the Supplemental materials.***

***English form was revised.***

***A synoptical scheme of the protocol was added in the Supplemental materials.***

***Update 14^th^ November 2021:***

1. Please amend the title either on the online submission form or in your manuscript so that they are identical.

***Answer: The title was amended on the manuscript, as requested, and now is identical to the title on the online submission form.***

2. Thank you for noting that your data will be made available. Please note that PLOS ONE’s policies on Data Availability require that all data necessary to replicate the findings of the manuscript be made available upon acceptance.PLOS ONE strongly recommends that this data be deposited to a stable public repository such as Figshare (http://figshare.com/) or Dryad (datadryad.org). For a list of other PLOS recommended repositories, please visit: <http://journals.plos.org/plosone/s/data-availability#loc-recommended-repositories>. Should your paper be accepted for publication, we will ask that you please provide a DOI or accession number for your dataset. We will then update the Data Availability statement associated with your manuscript on your behalf. Ensuring data deposition before acceptance of your manuscript will help to prevent delays in the acceptance process.

***Answer: At the moment a data repository is not yet available as we are still completing the data collection and cleaning. Data will be made available at the end of the project, after completing the study analyses. As the present article is a description of the study protocol, no findings to be replicated are now present.***
